# Prevalence of burnout in physiotherapists during the COVID-19 pandemic: systematic review and meta-analysis

**DOI:** 10.3389/fpubh.2026.1978417

**Published:** 2026-10-29

**Authors:** Matteo Paci, Nicholas Berbenni, Alessandro Ugolini, Lapo Bianchi, Lorenzo Chessa, Martina Giusti, Mauro Di Bari

**Affiliations:** 1 Department of Experimental and Clinical Medicine, University of Florence, Florence, Italy; 2 Department of Allied Health Professions, Azienda USL Toscana Centro, Florence, Italy; 3 Graduate Course in Rehabilitation Sciences, University of Florence, Florence, Italy; 4 Department of Allied Health Professions, Azienda Ospedaliero Universitaria Pisana, Pisa, Italy; 5 Escola Superior de Saúde Atlântica (ESSATLA), Oeiras, Portugal; 6 Department of Medicine and Geriatrics, Azienda Ospedaliero-Universitaria Careggi, Florence, Italy

**Keywords:** COVID-19, depersonalization, emotional exhaustion, personal accomplishment, physiotherapy, prevalence, professional burnout

## Abstract

This study aimed to obtain a pooled estimate of the prevalence of burnout in physiotherapists during the COVID-19 pandemic and to explore differences between low- and middle-income countries (LMICs) and high-income countries (HICs). A systematic search was conducted on six databases, from December 2019 to June 2026. Two reviewers independently extracted data from the included studies and assessed the risk of bias using a modified version of the Newcastle-Ottawa Scale. The pooled prevalence was calculated through meta-analysis of proportions with 95% confidence interval (CI). Prevalence of overall burnout and of its three dimensions of emotional exhaustion (EE), depersonalization (DP), and reduced personal accomplishment (PA) was estimated. A total of 23 studies was included and 19 was used for meta-analyses. Pooled prevalence (95% CI) estimate of 18% (9–35), higher than usually observed pre-COVID. The pooled prevalence of the three MBI components was: EE, 46% (35–57); DP, 46% (21–73), and low PA, 34% (13–64), and tended to be higher in LMICs, although the difference between LMICs and HICs were statistically significant only for EE. The findings indicate a substantial prevalence of burnout among physiotherapists during the COVID-19 pandemic. However, substantial heterogeneity and differences in burnout definitions across studies should be considered when interpreting these estimates. To prevent burnout, special attention should be given to healthcare professionals’ wellbeing during exceptional events such as the COVID-19 pandemic.

**Systematic review registration:**

PROSPERO, CRD420251029422.

## Introduction

Burnout is a psychological syndrome, described as a self-reported state of care- or work-related physical and mental stress (1). According to the Maslach Burnout Inventory (MBI), its components are emotional exhaustion (EE), depersonalization (DP), and a sense of reduced personal accomplishment (PA) (2). EE reflects a state of physical and emotional fatigue resulting from the depletion of an individual’s emotional energy. DP is characterized by the development of detached, negative, or cynical attitudes toward patients, leading to emotional distancing in professional interactions. Reduced PA refers to a diminished sense of competence and professional efficacy, often accompanied by low self-esteem and dissatisfaction with one’s work. Burnout has been recently recognized as an “occupational phenomenon” in the International Classification of Diseases 11th revision (ICD-11) by the World Health Organization (WHO) and a relevant health issue (3). As such, it leads to negative effects from the workers’ perspective and represents a concern for patient’s safety, as it might negatively impact on work performance and quality (4).

Burnout prevalence is influenced by multiple contextual factors, including the work environment, organizational characteristics, and broader cultural differences, which may contribute to variability across countries and healthcare settings. Recent evidence suggests that the determinants of burnout can be broadly classified into four domains: structural and organizational factors, psychological and emotional factors, environmental influences, and sociodemographic characteristics (5). Physiotherapists may be particularly vulnerable to burnout because their profession involves continuous, patient-centered care that requires close interpersonal relationships with patients and their caregivers. In many rehabilitation settings, especially long-term care facilities and the management of chronic conditions (6), therapeutic relationships often extend over prolonged periods, demanding sustained emotional engagement. At the same time, physiotherapists employed in acute care hospitals have also been shown to experience high levels of emotional exhaustion and depersonalization, reflecting the psychological burden associated with fast-paced and demanding clinical environments (7). In a recent meta-analysis, the pooled prevalence of burnout among physiotherapists was 8%, with figures of 27, 23, and 25% for the three dimensions of EE, DP, and PA included in the MBI (8). These findings were comparable to those obtained in physicians and other healthcare workers (9, 10).

The unprecedented global public health crisis, recently experienced worldwide with the Coronavirus Disease 2019 (COVID-19) pandemic [World Health Organization, (11)], imposed a significant burden on the health care system. In addition to the direct impact of COVID-19 on affected patients, the mental health of healthcare providers was severely challenged by the pandemic. Healthcare workers who had to be quarantined were at a greater risk of avoidance behaviors and more severe symptoms of post-traumatic stress disorder (PTSD) than the general population (12).

Previous systematic reviews with meta-analyses reported that the prevalence of burnout among healthcare professionals, mainly nurses and physicians, during COVID-19 pandemic was as high as 50% (13–16). Although the range of the reported prevalence figures across different studies was wide (4.3–90.4%) (17), there is a general consensus that the prevalence was higher during the pandemic than before (18).

As previously suggested (19), socioeconomic, organizational, and cultural differences between high income (HICs) and low- and middle-income countries (LMICs) may substantially influence both the occurrence and perception of burnout, resulting in marked variability in reported prevalence estimates. These differences may reflect disparities in healthcare resources, workforce capacity, occupational support, working conditions, and access to mental health services, all of which were further challenged during the COVID-19 pandemic.

Limited evidence, represented by a review without meta-analysis of only 4 studies (20), suggested higher prevalence during the pandemic than before also in physiotherapists. To consolidate this evidence, we conducted the present systematic review with meta-analysis, whose primary aim was to estimate the prevalence of burnout among physiotherapists during the COVID-19 pandemic. Moreover, because the prevalence of burnout during the pandemic was shown to be greater in HICs than in LMICs (14, 21), we aimed also exploring differences in physiotherapists’ burnout between LMICs and HICs.

## Methods

The protocol and details were registered into the International Prospective Register of Systematic Reviews - PROSPERO (CRD420251029422). The reporting of this systematic review follows the PRISMA guidelines (22).

PubMed, CINAHL, Web of Science, Embase, Scopus and PsycINFO were searched, from December 2019 to June 2026 (last search June 20, 2026), to retrieve original studies, reporting the prevalence of burnout among physiotherapists related to pandemic period. Full search string is reported in Appendix A.

In addition, reference lists of related articles were checked by two independent reviewers to ensure the retrieval of all potentially relevant publications. Two independent reviewers (NB and LC) excluded non-pertinent articles after reading the title and the abstract, then retrieved and assessed for inclusion the full text. Disagreement was resolved by consensus and, where consensus could not be reached, by a third reviewer (MP). Studies were included when they: (1) investigated the prevalence of burnout among physiotherapists in relation to the COVID-19 pandemic; (2) when involving different categories of healthcare professionals, reported separately data specifically referring to physiotherapists. Professionals delivering respiratory physiotherapy under a distinct professional title were considered physiotherapists, given the functional equivalence of their clinical role. Physiotherapist assistants analyzed jointly with physiotherapists were also included, as separate data were not available; (3) were published in English, Spanish, Portuguese, Italian, French, or German. Studies on physiotherapy students, abstracts from conference proceedings, and qualitative studies were excluded. When the method of assessment of burnout was not explicitly reported, it was inferred based on the articles or manuals cited in the study, when reported. When no diagnostic cutoff was explicitly reported, it was inferred based on the articles or manuals the studies cited, when available.

### Risk of bias assessment

Two independent reviewers (NB and LB) assessed the risk of bias using a modified version of the Newcastle-Ottawa Scale (23); Disagreements were resolved by consensus and, where consensus could not be reached, by a third reviewer (MP). The reviewers were physiotherapists with experience in the methodological evaluation of studies. A one-hour meeting was nevertheless held to define the assessment criteria for the scale. The third reviewer was a senior physiotherapist with extensive experience in assessing articles. Each item in the checklist was scored as “1 = yes” or “0 = no”; the global score, obtained by summation, quantified the methodological quality of each paper. The tool, which can be used to assess both cross-sectional and cohort studies, includes three areas of evaluation: participants’ selection (‘Selection’, subdivided into three subitems that can be assigned a score of 0 or 1); comparison of subjects (‘Comparability’, subdivided into two subitems that can be assigned a score of 0 or 1); outcome (two subitems that can be assigned scores of 0, 1 or 2 and 0 or 1, respectively). Based on the summary score, a study can be defined as at a low, medium, or high risk of bias when scoring 7 or 8, 6, and less than 6, respectively.

### Statistical analysis

When burnout was reported as an ordinal variable, the prevalence estimate was derived according to the case definition reported in the original study. When the study defined burnout as a combination of moderate and high categories, these categories were combined. When only the highest severity category was defined as the outcome of interest, the reported proportion of participants in that category was used. For the personal accomplishment dimension, the direction of the scale was considered, with low personal accomplishment representing the adverse outcome. The case definition and the categories included in the prevalence estimate were recorded for each study and outcome to ensure transparency regarding the criteria used for pooling. When different types of burnout (e.g., personal-, social- or work-related burnout) were reported, the prevalence of work-related burnout was considered. For longitudinal studies, the proportion of physiotherapists with burnout at any assessment during the pandemic was used.

Because the included studies used different instruments and operational definitions of burnout, pooled estimates were interpreted as prevalence estimates based on the case definitions adopted by the original studies rather than as estimates based on a single standardized diagnostic criterion.

A generalized linear mixed model (GLMM), specifically a random intercept logistic regression model, was used for the meta-analysis of proportions. Prevalence point estimates, together with their 95% confidence interval (CI), were reported in forest plots. Pooled estimates were obtained using fixed or random effects models, depending on the level of heterogeneity, which was quantified with the *I*^2^ index and tested with the Q statistics.

Subgroup analyses were performed according to the World Bank income classification (obtained International Statistical Institute: https://isi-web.org/low-and-middle-income-countries-and-regions) of the country in which the survey was conducted. Pooled prevalence estimates and 95% confidence intervals were calculated separately for LMICs and HICs. Differences between subgroups were assessed using the test for subgroup differences implemented in the meta-analysis model. The total score of the risk of bias assessment of studies conducted in LMICs and HICs were compared using the Mann–Whitney test.

In addition, meta-regressions were conducted considering potential moderators, such as the risk of bias, as assessed from the Newcastle-Ottawa Scale scores, and the year of publication of the article.

To provide a contextual comparison with pre-pandemic evidence, the pooled prevalence estimates obtained in the present review were compared descriptively with estimates reported in a previous meta-analysis conducted before the COVID-19 pandemic. This comparison was considered contextual rather than a direct comparison between the same populations or studies and was therefore interpreted cautiously.

All analyses were performed using Jamovi (The jamovi project, 2022; R Core Team, 2021) and the software R and the ‘meta’ package. Statistical significance was set at a *p*-value < 0.05 level.

## Results

The search provided a total of 2,864 titles, after the removal of 656 duplicates. After removing titles based on title and abstract (*n* = 2,805), 59 studies were screened. Of them, 41 were excluded after reading the full text, leaving 18 studies, which, together with the 5 studies identified by other methods, result in a total of 23 studies (24–46) available for the review (Figure 1). The articles included, thirteen conducted in HICs and 10 in LMICs, were published from 2020 to 2026 and involved from 2 to 671 participants (mean: 158.9 ± 192.3), total 3,655 participants Their main characteristics are reported in Table 1.

**Figure 1:**
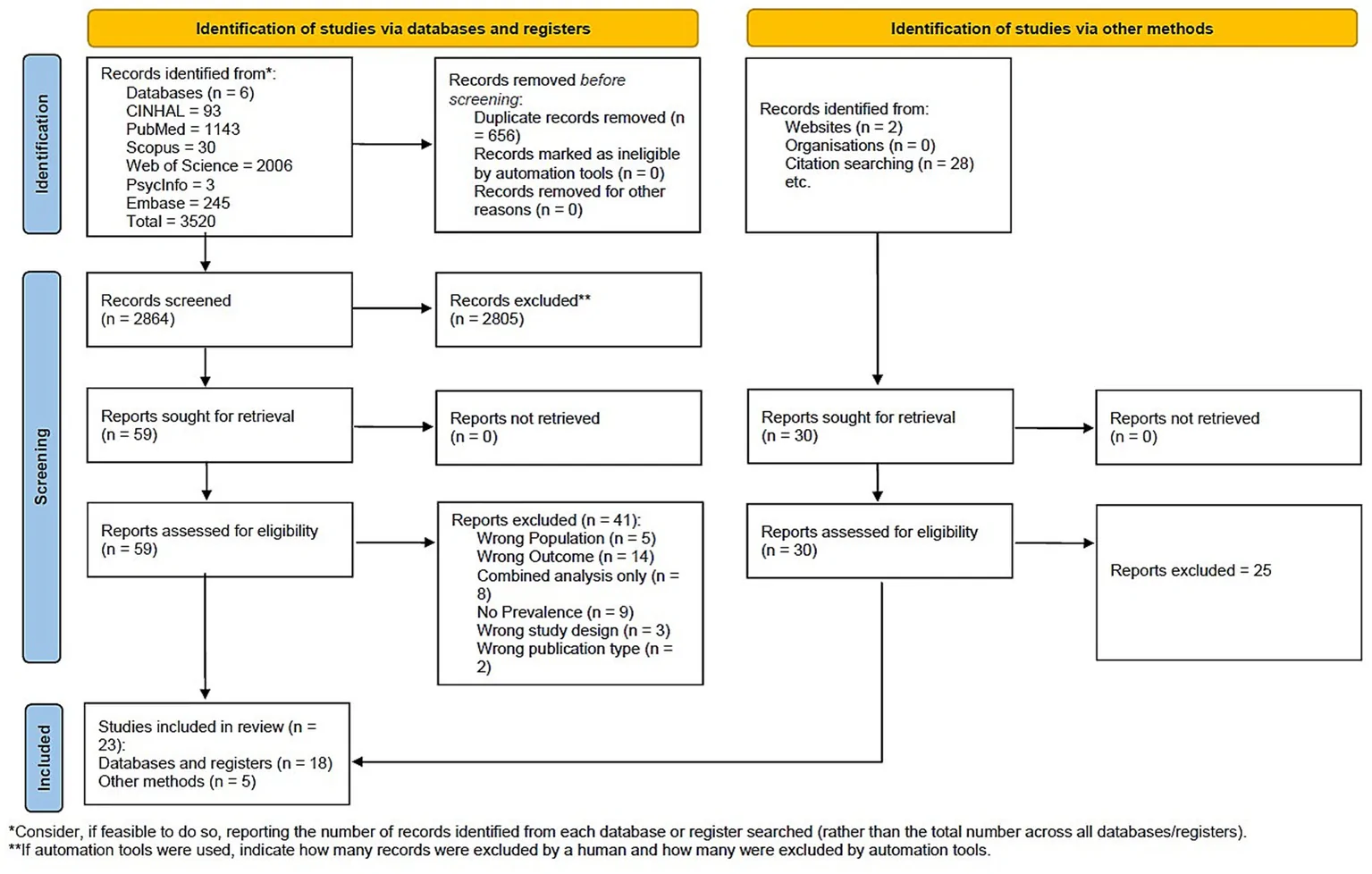
PRISMA flowchart of the search strategy of the studies included in the systematic review.

**Table 1:** Characteristics of included studies.

Source	Country	Income	Setting	No. Physiotherapists	Age	Gender (%)	Burnout assessment instrument	Overall burnout definition	Burnout dimension definitions	Overall prevalence (%)	Dimensions prevalence (%)
(24)	Peru	Low/Middle	Ministry of health, Social Insurance Institution, Military	57	39.2 ± 10.9	Females = 77.2 Males = 22.8	MBI	NR	High EE High DP low PA	1.8	High EE = 28.1 High DP = 19.3 Low PA = 12.3
(25)	Italy	High	clinics/practices, hospitals	671	41.1 ± 11.6	Females = 69.4 Males = 30.6	MBI-HSS (Italian Version)	Presence of Burnout: High EE ≥ 23 + High DP ≥ 6; Low PA ≤ 31	High EE High DP low PA	24.6	High EE = 40.2 High DP = 35.6 Low PA = 19.1
(26)	United States	High	State of Mississippi	187 (122 PT + 65 PTA)	<35 years = 40%; 35–44 years = 24%; 45–54 years = 22%; ≥55 years = 13%	Females = 67.0 Male = 33.0	ProQOL	Low ≤ 22; Medium 23–41; High ≥ 42	Compassion Satisfaction (CS); Secondary Traumatic Stress (STS); Burnout (BO)	Low burnout = 41.2; Medium burnout = 58.8; High burnout = 0.0	NR
(41)	Brazil	Low/Middle	Intensive care	136	37.9 ± 8.2	Females = 75.0 Males = 25.0	*Ad hoc* questionnaire	0–15 No Burnout; 16–30 Possibility of developing Burnout; 31–45 Initial stage of Burnout; 46–60 Presence of Burnout; 61–75 Advanced stage of Burnout	Physical exhaustion, Emotional exhaustion, Professional exhaustion; Depression and irritability	27.2	NR
(46)	Brazil	Low/Middle	Private and public clinics, hospitals	43	Clinics = 30.9 ± 6.5; Hospitals = 27.8 ± 3.6	Clinics (40): Females = 83.3 Males = 16.7 Hospitals (31): Females = 61.5 Males = 38.5	MBI-HSS	EE: Low ≤ 18; Medium 19–26; High ≥ 27; DP Low ≤5 Medium 6–9 High ≥ 10; PA Low ≤ 33 Medium 34–39 High ≥ 40	High EE High DP Low PA	NR	Clinics: High EE = 56.7 High DP = 93.3 Low PA = 96.7 Hospitals: High EE = 61.5 High DP = 100.0 Low PA = 100.0
(28)	United States	High	2 health systems	202 (PT + PTA)	Median = 34 (Range 24–62)	Females = 66.0 Males = 32.0 Intersex = 1.0 Not answered = 1.0	MBI-HSS	EE: Low ≤ 17; Medium 18–29; High ≥ 30; DP Low ≤5 Medium 6–11 High ≥12; PA Low ≤ 33 Medium 34–39 High ≥ 40	High EE High DP Low PA	NR	High EE = 27.6 High DP = 22.1; Low PA = 36.6
(29)	Colombia	Low/Middle	Private University hospital	31 (6 PT + 25 RT)	36.0 ± 8.7	Females = 93.5 Males = 6.5	MBI-HSS	EE: Low ≤18; Medium 19–26; High ≥ 27; DP Low ≤ 5 Medium 6–9 High ≥ 10; PA Low ≤ 33 Medium 34–39 High ≥ 40	Burnout = ≥1 dimension (High EE High DP Low PA)	38.7	High EE = 38.7 High DP = 16.1 Low PA = 3.2
(30)	United States	High	1 University trauma clinic	53 (PT + PTA)	NR	NR	ProQOL	BO: Low ≤ 22; Medium 23–41; High ≥ 42	Compassion Satisfaction (CS); Secondary Traumatic Stress (STS); Burnout (BO)	High Burnout = 0.0; Medium burnout = 60.4; Low burnout = 39.6	NR
(31)	Belgium	High	12 General hospitals	115	20–29 = 28.1%; 30–39 = 35.1%; 40–49 = 12.3%; ≥50 = 24.6%	Females = 55.7 Males = 44.3	PFI	Burnout ≥1.33; Professional fulfillment ≥3	Professional fulfilment; Professional exhaustion	33.9	NR
(32)	Portugal	High	Private clinics (50%) Wards (35%) Intensive care Units (14%) Continuous care/residential homes/day-care (10%) Primary care (7%) Palliative care (3%) Others (9%)	511	Median = 33 (IQR = 28;41)	Females = 81.6 Males = 18.4	CBI	Personal Burnout ≥50; Work-related Burnout ≥50; Patient-related Burnout ≥50	Personal Burnout; Work-related Burnout; Patient-related Burnout	42.1	Personal Burnout = 41.7; Work-related Burnout = 42.1; Patient-related Burnout = 25.4
(34)	United Arab Emirates	High	Rehabilitation Clinics	47	20–29 = 10 (11.9%); 30–39 = 53 (63.1%); 40–49 = 21 (25.0%)**	Females = 69.0 Males = 31.0**	MBI-HSS	EE: Low ≤18; Medium 19–26; High ≥ 27; DP Low ≤ 5 Medium 6–9 High ≥ 10; PA Low ≤ 33 Medium 34–39 High ≥ 40	High EE High DP Low PA	23.4	High EE = 25.5 High DP = 19.1 Low PA = 59.6
(33)	Greece	High	2 Primary Health Care Public Hospitals	48	25–30 = 2.1%; 31–45 = 33.3%: 46–55 = 45.8%; 56–60 = 18.8%	Female = 30 Male = 18	MBI	NR (no cut-off based)	High EE High DP Low PA	50.0	High EE = NR High DP = 39.6 Low PA = 31.3
(35)	Brazil	Low/Middle	29 Paediatric intensive care units (13 public; 16 private)	147^a^	NR	NR	MBI-HSS	≥1 dimension at high risk: High EE > 26; High DP > 9; Low PA < 33	High EE High DP Low PA	17.0^a^	NR
(36)	Japan	High	1 Tertiary care centre	13	NR	NR	MBI-GS	High EE ≥ 3.5; Cynicism ≥ 3.5; Professional efficacy ≤ 2.5	Exhaustion; Cynicism; Professional efficacy	0.0	NR
(37)	Brazil	Low/Middle	6 public Intensive care Units	80	NR	NR	MBI-HSS	High EE ≥ 26; High DP ≥ 9; Low PA ≤ 33	High EE High DP Low PA	NR	High EE = 58.8 High DP = 27.5 Low PA = 16.3
(27)	Brazil	Low/Middle	Intensive care	67	Mean = 31 (Range = 22–50)	Females = 52.2 Males = 47.8	MBI	NR	High EE High DP Low PA	7.5^b^	High EE = 67.2 High DP = 94.0 Low PA = 6.0
(38)	Greece-Cyprus	High	Private and public Healthcare centres	163 (159 PT + 4PTA)	NR	NR	CBI	<50 = No Burnout; 50 to <75 = Moderate BO; 75 to <100 = High Burnout; 100 = Severe Burnout	Personal Burnout; Work-related Burnout; Patient-related Burnout	53.4	NR
(39)	United States	High	Private and Public centres	420	Mean = 29.1 ± 3.2	Females = 72.1; Males = 26.4; Not Answered = 0.7; Other = 0.7	PFI	Professional Burnout ≥1.33	Interpersonal disengagement; Professional exhaustion	67.9	NR
(40)	Japan	High	487 Rehabilitation Clinics	566	Median = 37 (IQR = 31–45)	Females = 21.2 Males = 78.8	MBI-GS	High EE > 3.5 Cynicism > 3.5; Professional efficacy < 2.5	Exhaustion; Cynicism; Professional efficacy	17.5	NR
(42)	Ireland	High	2 Emergency departments	2	NR	NR	OLBI	Total score ≥2.18	Disengagement; Exhaustion	50.0	NR
(43)	India	Low/Middle	Healthcare centres	74	NR	NR	CBI	<50 = No Burnout; 50 to <75 = Moderate BO; 75 to <100 = High Burnout; 100 = Severe Burnout	Personal Burnout; Work-related Burnout; Patient-related Burnout	75.7	Personal Burnout = 36.5; Work-related Burnout = 75.7; Patient-related Burnout = 58.1
(44)	Brazil	Low/Middle	1 Tertiary care centre	12^c^	NR	NR	OLBI	Disengagement ≥ 2.1 & Exhaustion ≥ 2.25	Disengagement; Exhaustion	41.7^c^	NR
(45)	Ecuador	Low/Middle	Intensive care	10	NR	NR	MBI	NR	High EE High DP Low PA	10.0	High EE = 80.0 High DP = 10.0 Low PA = 20.0

**Data from the whole healthcare sample.

a Physiotherapists with complete MBI data; n derived from the reported cases (17%).

b Self-reported physician diagnosis of burnout; not derived from MBI.

c Period prevalence: physiotherapists with burnout (*n* = 2) at baseline plus incident cases at fol*l*ow-up (*n* = 3); 5/12.

### Assessment tools

In the included studies, 6 different instruments were used to assess burnout: the MBI, the Oldenburg Burnout Inventory (OLBI), the Copenhagen Burnout Inventory (CBI), the Professional Quality of Life, the Stanford Professional Fulfillment Index (PFI) and a questionnaire developed by the authors.

The most frequently used assessment tool was the MBI, which was present in 13 studies (24, 25, 27–29, 33–37, 40, 45, 46). Of these, the General Survey version was used in two studies (36, 40) and the Human Service Survey version in 7 studies (25, 28, 29, 34, 35, 37, 46). In 4 studies the authors reported only using the MBI, without specifying the version of the tool (24, 27, 33, 45). The CBI and the ProQOL were used in three (32, 38, 43) and two studies (26, 30), respectively. The PFI (31, 39) and OLBI (42, 44) were used in two studies while Moura et al. (41) used an *ad hoc* questionnaire.

### Risk of bias

As shown in Table 2, the quality assessment score ranged from 2 to 6 points (median = 5; interquartile range = 4–6) across the studies included and no difference between LMICs and HICs at the Mann–Whitney test (*p* = 0.820) was found. No studies were found to have a low risk of bias, 9 studies received a score of 6, corresponding to a moderate risk of bias, while the remaining 14 studies received a score ≤5, indicating a high risk of bias. All the studies used a valid measure to detect burnout and its components, although with different cut-offs across studies, while only one study assessed comparability between subjects who did or did not participate.

**Table 2:** The quality assessments of included studies.

Source	Item 1	Item 2	Item 3	Item 4	Item 5	Item 6	Item 7	Total
(24)	1	0	0	1	1	1	1	5
(25)	0	1	0	1	1	1	1	5
(26)	1	0	0	1	1	1	1	5
(41)	1	1	0	1	1	1	1	6
(46)	0	0	0	1	1	1	0	3
(28)	1	1	0	1	1	1	1	6
(29)	1	1	0	1	1	1	1	6
(30)	1	0	0	0	0	1	0	2
(31)	1	0	1	1	1	1	1	6
(32)	1	1	0	1	1	1	1	6
(33)	1	0	0	1	1	1	0	4
(34)	1	0	0	1	1	1	1	5
(35)	1	1	0	1	1	1	1	6
(36)	1	0	0	1	1	1	1	5
(42)	0	0	0	1	1	1	1	4
(37)	1	1	0	1	1	1	1	6
(27)	0	0	0	1	0	1	1	3
(38)	0	0	0	1	1	1	1	4
(39)	1	1	0	1	1	1	1	6
(40)	1	1	0	1	1	1	1	6
(43)	1	0	0	1	1	1	1	5
(44)	0	0	0	1	1	1	1	4
(45)	1	0	0	1	1	1	0	4

Item 1: Representativeness of the sample; Item 2: Sample size; Item 3: Non-included subjects; Item 4: Comparability 1; Item 5: Comparability 2; Item 6: Assessment of the outcome; Item 7: Statistical test.

### Meta-analyses

Overall burnout, estimated from 19 studies, had a pooled prevalence (95% CI) of 18% (9–35) with substantial heterogeneity (*I*^2^ = 95.6%), within a range between 0 and 76% (Figure 2). Differences between LMICs and HICs were not statistically significant for overall burnout (Figure 3). Heterogeneity remained high in all analyses, with *I*^2^ values of 92.4 and 96.8%, respectively.

**Figure 2:**
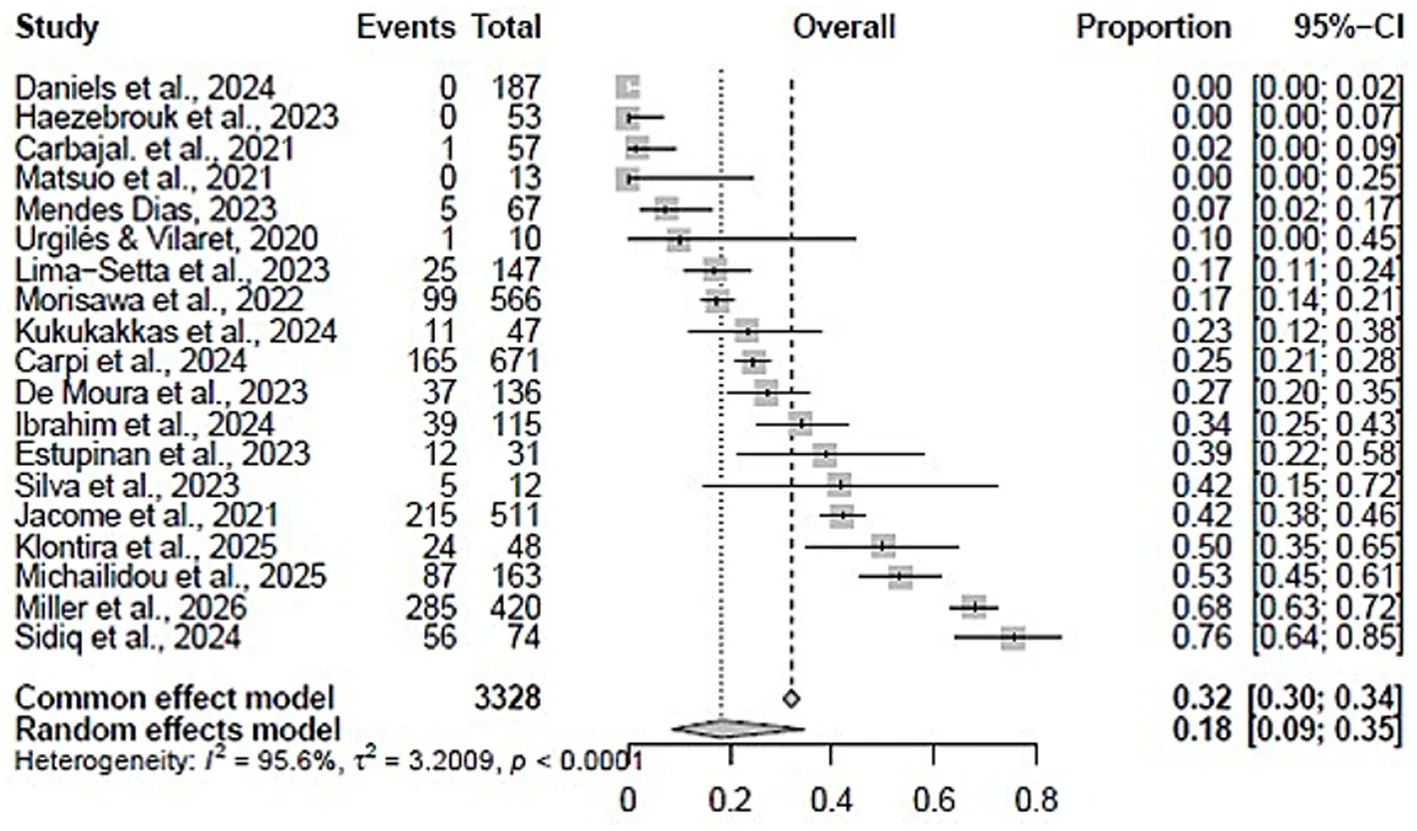
Pooled overall prevalence of burnout across all studies.

**Figure 3:**
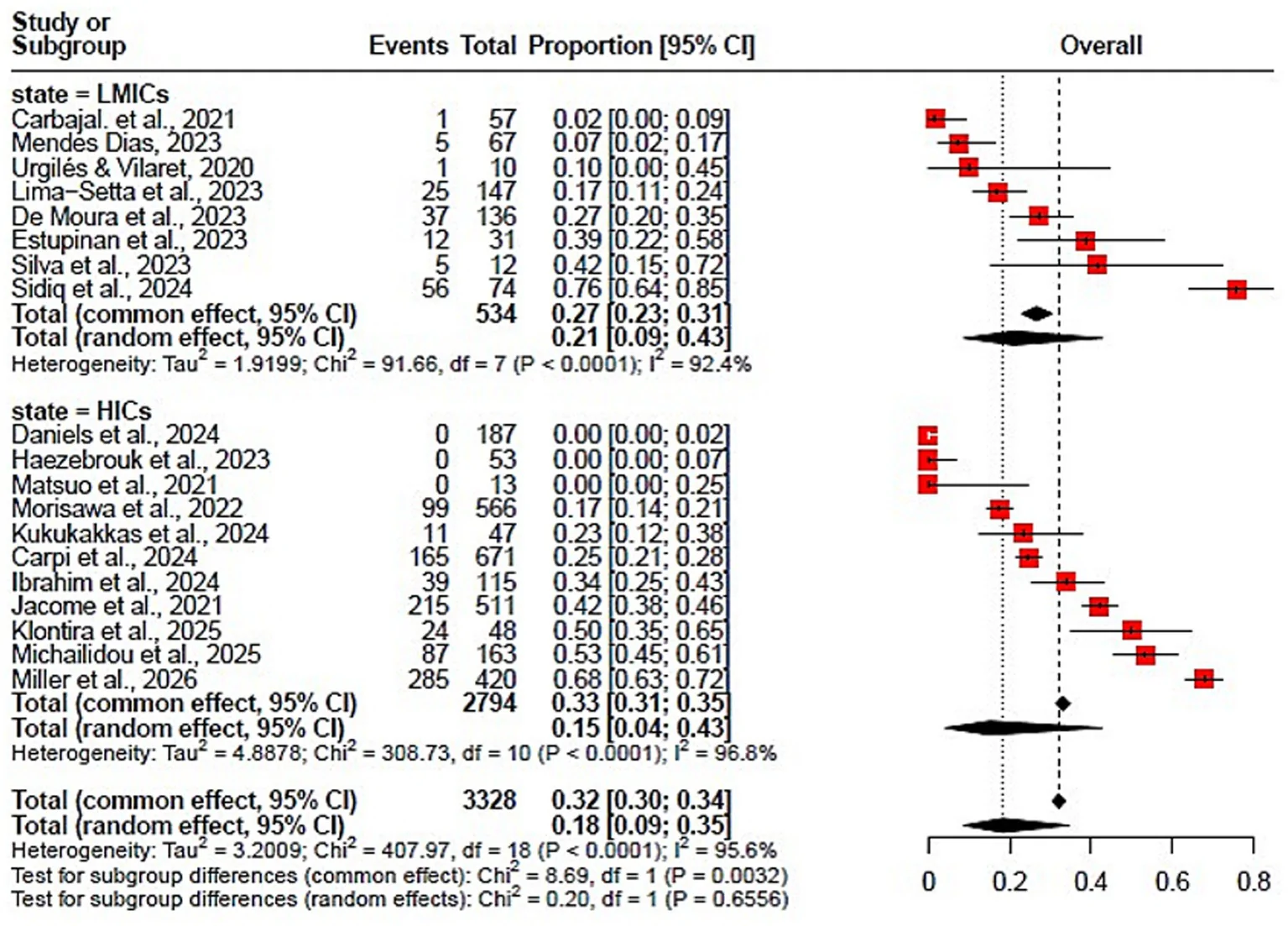
Subgroup analyses. Pooled overall prevalence of burnout from studies conducted in low- and middle-income countries and high-income countries.

### Maslach burnout inventory dimensions

The pooled (95% CI) prevalence for the three dimensions of MBI, reported in 10 studies, except for EE reported in 9 studies, was 46% (35–57) for EE (Figure 4), 46% (21–73) for low DP (Figure 5), and 34% (13–64) for low PA (Figure 6), respectively. Heterogeneity was high in all analyses, with *I*^2^ values ranging from 85.0 to 89.5%. The prevalence of burnout is lower in HICs than in LMICs only for EE (Figure 7) component, not DP (Figure 8) and PA (Figure 9). Heterogeneity was high in all analyses, with *I*^2^ values ranging from 75.8 to 94.2%.

**Figure 4:**
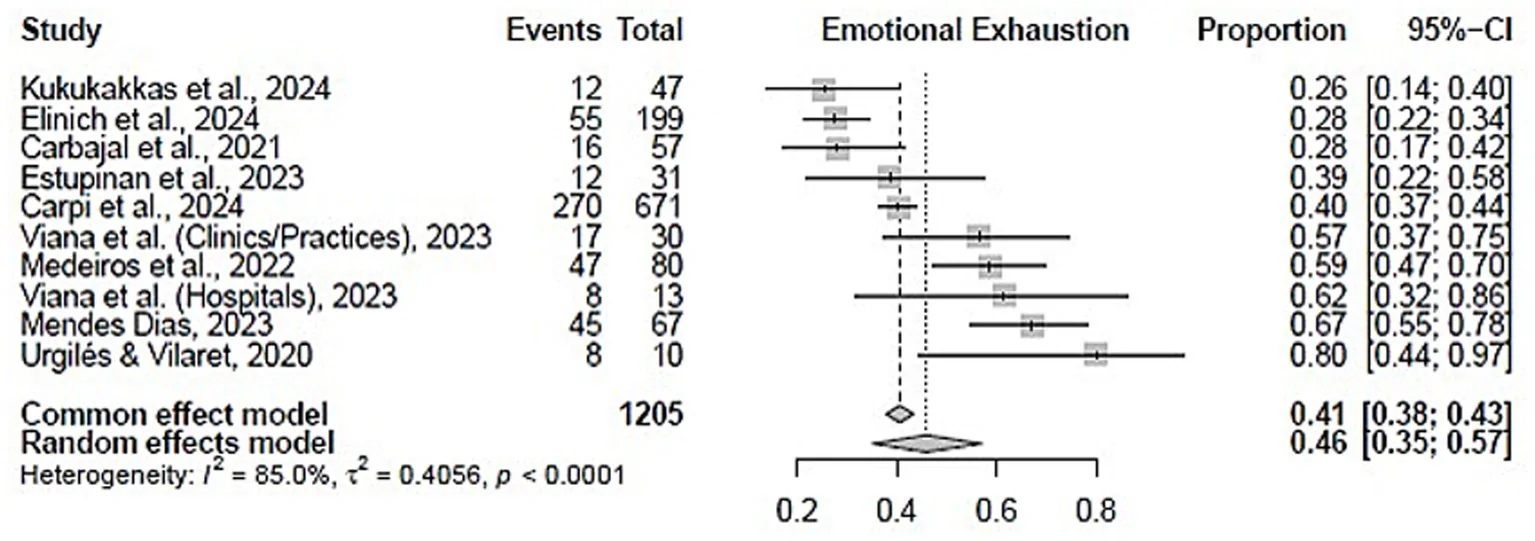
Pooled prevalence of Emotional Exhaustion dimension across all studies.

**Figure 5:**
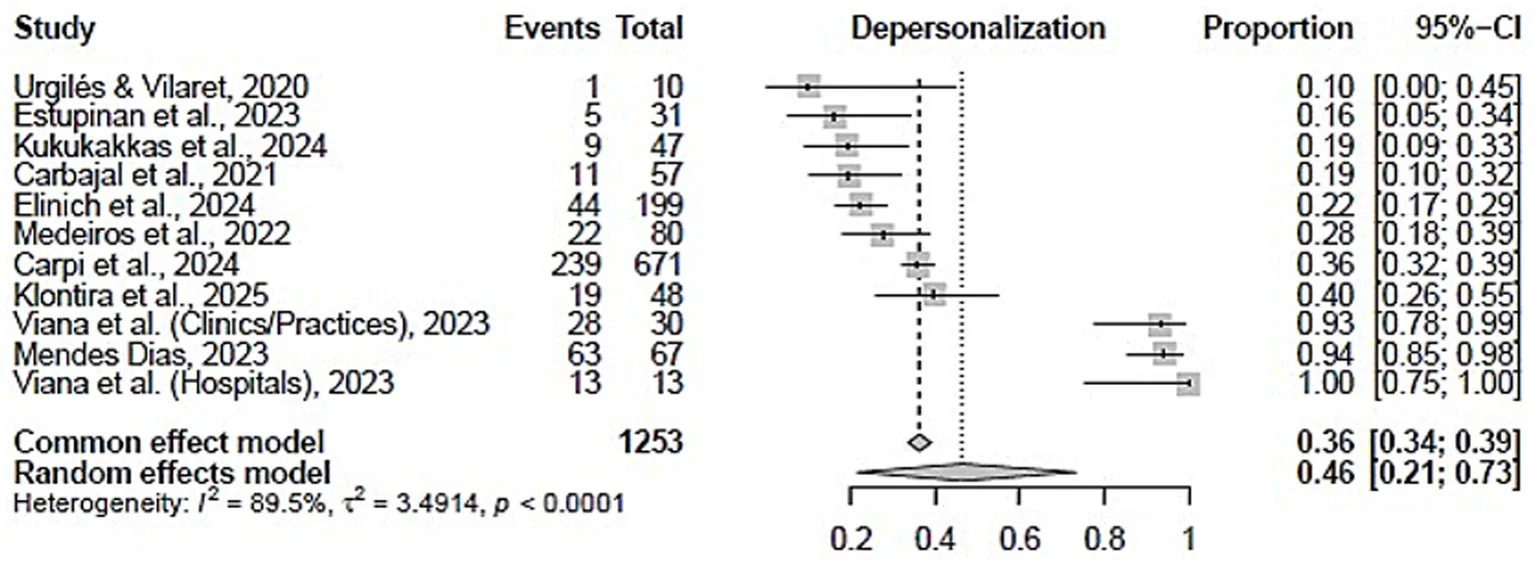
Pooled prevalence of Depersonalization dimension across all studies.

**Figure 6:**
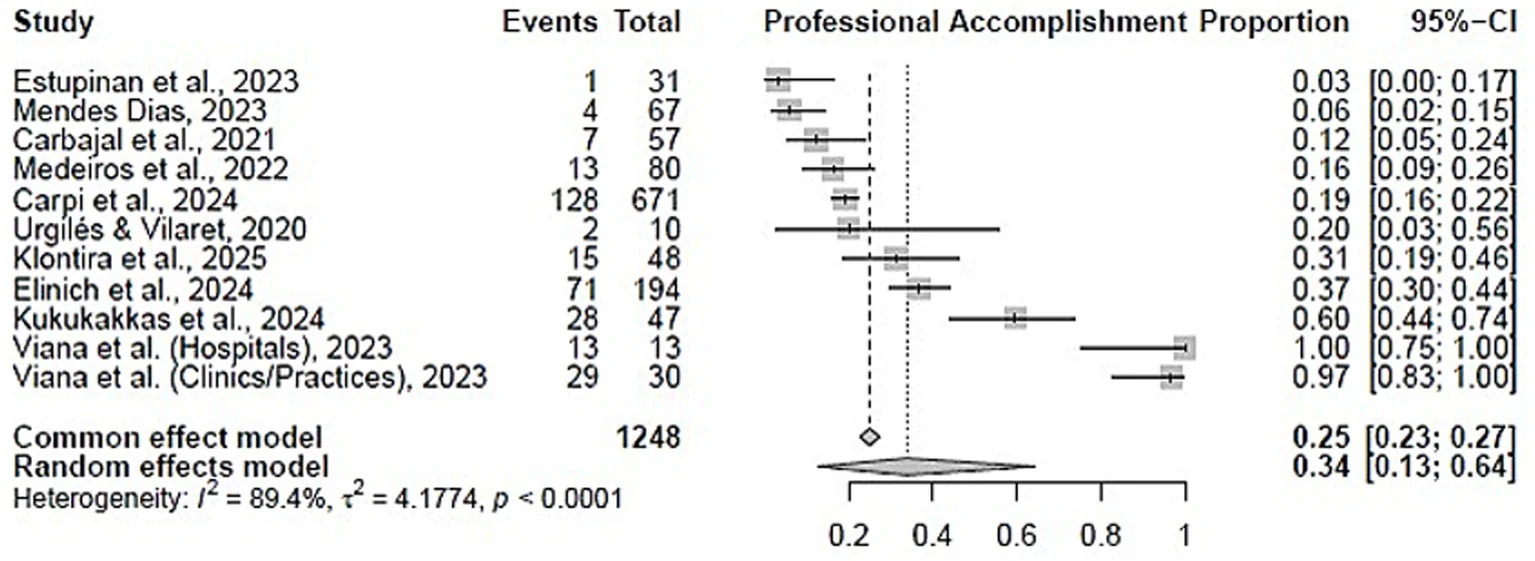
Pooled prevalence of Professional Accomplishment dimension across all studies.

**Figure 7:**
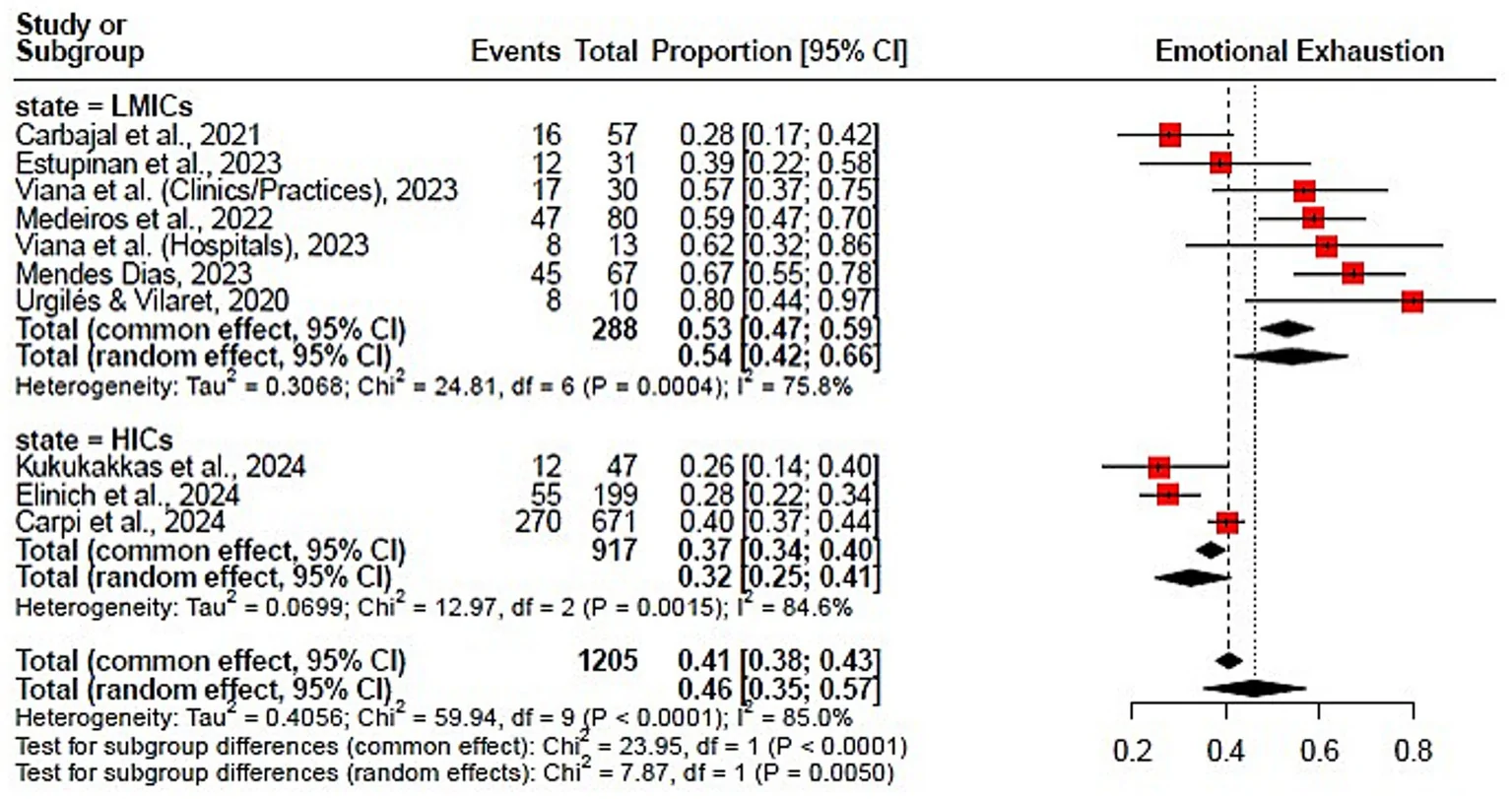
Subgroup analyses. Pooled prevalence of Emotional Exhaustion dimension from studies conducted in low- and middle-income countries and high-income countries.

**Figure 8:**
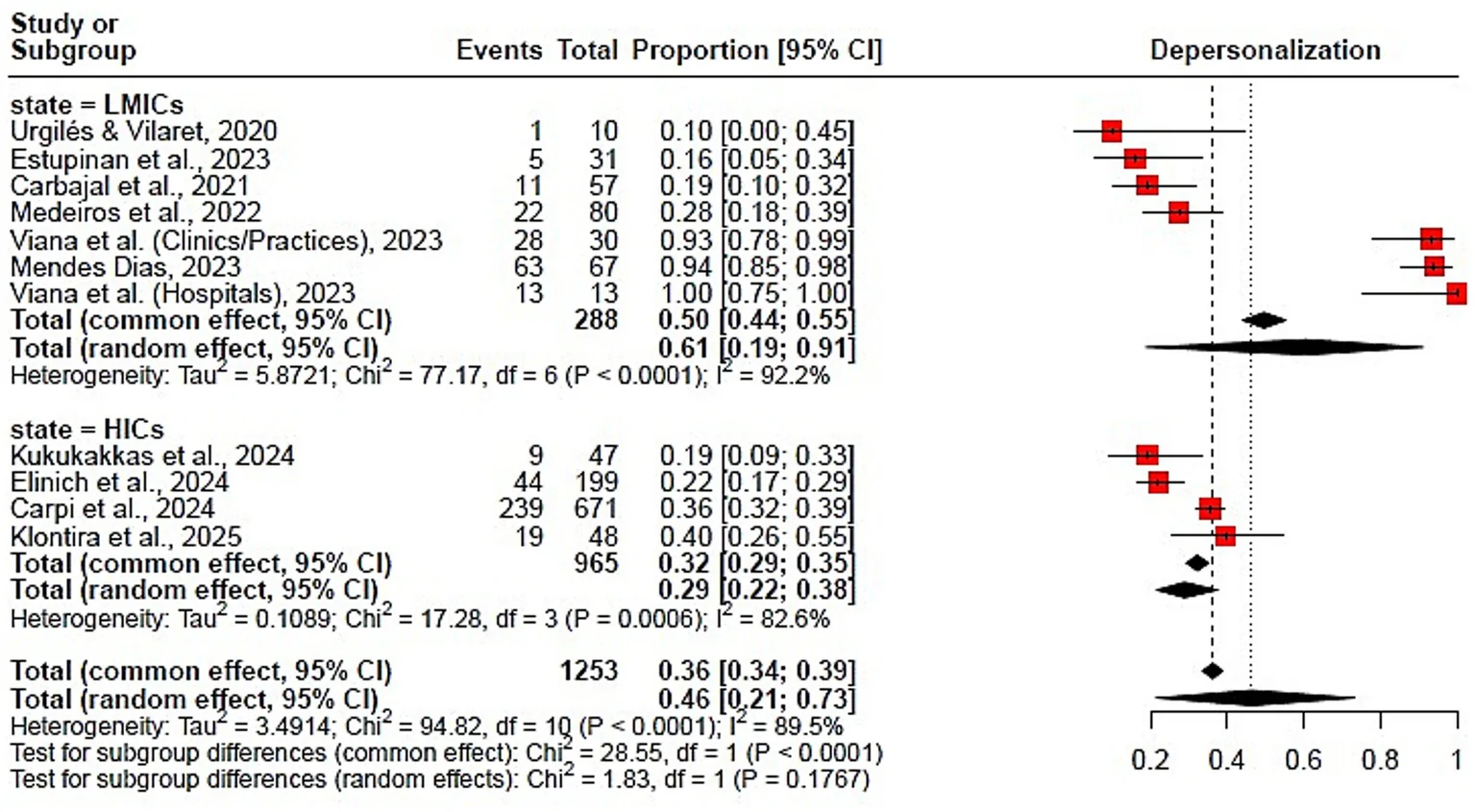
Subgroup analyses. Pooled prevalence of Depersonalization dimension from studies conducted in low- and middle-income countries and high-income countries.

**Figure 9:**
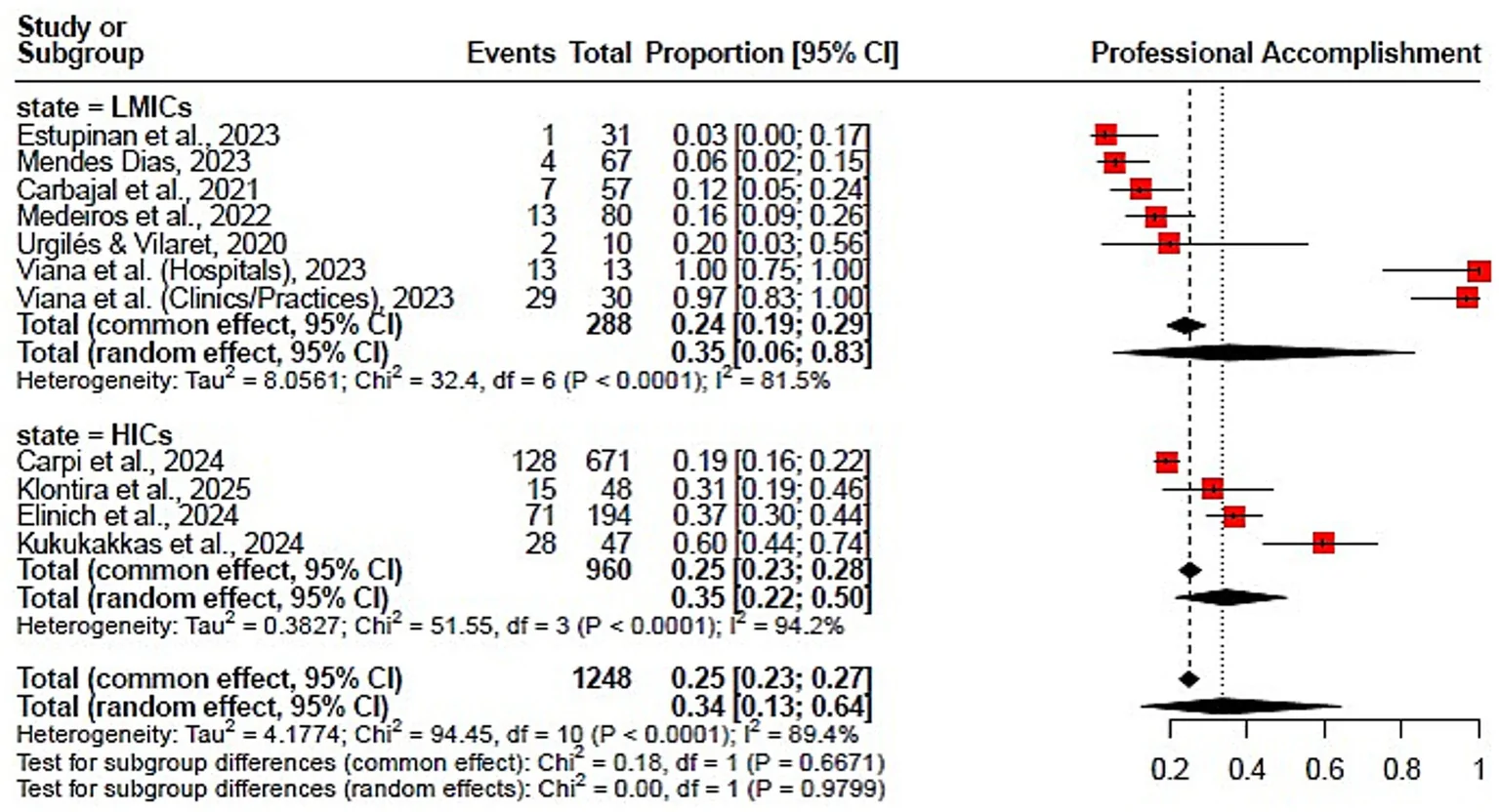
Subgroup analyses. Pooled prevalence of Professional Accomplishment dimension from studies conducted in low- and middle-income countries and high-income countries.

Meta-regressions, also reported in Appendix B, suggested that the variables considered did not contribute substantially to the high heterogeneity. However, two significant moderators were found that only partially explained the high heterogeneity. For the outcome overall burnout, the year of the study was a significant moderator (*p* < 0.05) with a positive effect. The quality of the studies was a moderator for EE (<0.05) and D (*p* < 0.001) with a negative effect. No other moderator achieved statistical significance.

## Discussion

To our knowledge, this is the first meta-analysis on the prevalence of burnout among physiotherapists during the COVID-19 pandemic. The pooled prevalence of overall burnout was 18% (9–35). In subgroup analyses, burnout prevalence tends to be higher in LMICs than in HICs, although statistically significant difference between LMICs and HICs was found only for EE. Burnout has been a relevant health issue among physiotherapists during the pandemic, because as many as 23 studies from 14 countries were found, all conducted in a relatively short timeframe.

Consistent with the results of Ghahramani et al. (14), low PA was the least prevalent of the three dimensions of burnout. A possible explanation is that, although the increase of the workload for healthcare professionals during the COVID-19 pandemic, they may feel more useful and valued by the community, which could lead to an increase in PA (14). However, these aspects are not sufficient to prevent the higher prevalence found. Some factors, such as organizational settings, work environment and climate, and support by managers should be given special attention during exceptional events such as a COVID-19 pandemic (40, 47).

The pooled prevalence of 18% was more than two times as high as the 8% value (Appendix C), reported in a meta-analysis referring to pre-pandemic years (8). This comparison provides contextual evidence suggesting a greater burden of burnout during the pandemic period; however, it should not be interpreted as a direct estimate of the effect of the COVID-19 pandemic, because the two estimates derive from different sets of studies, populations, instruments, and study periods.

These data are consistent with the suggestion proposed in a recent systematic review (20). However, our analysis was performed using the same methodological approach and comparable outcome definitions, thereby improving the methodological comparability of the two estimates. Although the two reviews do not provide a direct comparison of the same populations or primary studies, the consistency of the analytical approach strengthens the value of this comparison as an indication of a potentially greater burden of burnout during the COVID-19 period. This finding should nevertheless be interpreted cautiously, as differences in study populations, settings, and timing of assessment may also contribute to the observed difference. Similar results have been found in a study on Spanish radiologists (18), which compared the prevalence during COVID with a previous survey of the same population. Conversely, Shanafelt (48) reported an improvement in burnout among U.S. physicians before and after the pandemic, which was attributed to political and organizational efforts to improve the health care delivery.

Possible explanations can be traced back to the unpredictable and potentially dangerous characteristics of COVID-19 and included: having to face a new, often life-threatening disease, with poor knowledge of its characteristics and treatment strategies; feeling frustrated because the prognosis of so many critical patients remained ominous, no matter how many efforts might be dedicated to them; warring for personal safety before such a highly contagious and severe disease. These negative feelings were probably more intense and pervasive in those professionals who were diverted to acute care wards from other services that, conversely, were dramatically downsized. Additional factors that might have increased burnout perceptions included lack of peer interaction and supervisors’ support, changes in work environment and organization, and reduced training opportunities (40, 47). Other risk factors reported as contributing to an increased risk of burnout during the COVID-19 pandemic include inadequate preparedness—both at hospital and community level, whether actual or perceived—limited availability of personal protective equipment, and shortcomings relating to the ethical climate, institutional protocols and organisational communication (13). For overall burnout, EE, D, and PA, the subgroup analyses did not provide evidence of statistically significant differences between LMICs and HICs, except for higher prevalence of EE in the LMICs group of studies. This finding should be interpreted cautiously, as country-income classification may capture a broader set of contextual differences, including healthcare system characteristics, working conditions, organizational resources, workload, and professional roles. Moreover, the number and geographical distribution of studies from LMICs were limited, which may affect the robustness and generalizability of this comparison. In fact, different findings on overall burnout have been reported by Ghahramani et al. (14), who reported higher COVID-19 burden in HICs, early on during the pandemic. In addition, greater experience of working in conditions of high need and limited availability of supplies would reduce burnout among health workers in LMICs, as suggested by Morgantini et al. (21).

The estimated prevalence of burnout appeared to be lower than that reported in mixed health care populations, especially nurses and physicians (14–16). The reasons why physiotherapists seem to be less vulnerable to burnout needs further study: it might be speculated that this finding is associated with a lower time of exposure to the disease and death, a lower level of direct responsibility for patient’s survival, the lack of an extra workload associated with 24-h availability, and the need to provide care that could not be postponed, especially when physiotherapists were not involved in the acute phases of the disease. In any case, many factors can have contributed to burnout among healthcare professionals during the pandemic, with some professionals experiencing symptoms of burnout more than others (17).

It should also be noted that, due to the smaller number of studies included, the 95% CI were wider than in the previous review (8). Thus, the lack of credible associations should be taken with some caution.

High heterogeneity observed in the studies included was expected, since it has also been found in other meta-analyses on different populations (14–16). It could be due to several sources, such as the tools used to assess burnout, the cut-off scores chosen to identify burnout prevalence, and the different settings and clinical experiences within and between studies. Other differences could be attributed to cultural contexts and healthcare systems variability among studies (15). The inclusion of studies from different or non-differentiated pandemic periods among countries can also contribute to the heterogeneity.

Another source of heterogeneity may be differences in the definition of burnout and the conceptual framework of its construct. The included studies used different instruments, cut-off values, and combinations of severity categories. Consequently, the pooled estimates should not be interpreted as representing a single standardized threshold of burnout severity. This methodological variability may have contributed substantially to the wide confidence intervals and high between-study heterogeneity observed in the meta-analyses. Some authors (49, 50) have also questioned the appropriateness of meta-estimating the prevalence of burnout because the scores obtained with the MBI cannot be used as diagnostic criteria. However, according to other authors, the dichotomization or combination of the subscales for the diagnosis of burnout is still useful for the identification of burnout (51). In fact, for example, the three dimensions of the MBI (EE, DP, PA) were differently defined by different authors: while Kucukakkas et al. (34) set the score needed to define “high” risk of burnout at 27, Elinich et al. (28) set the same threshold at 30. In addition, other authors reported the cut-off values needed to define “high” risk, but not those needed to define “medium” and “low” risk.

## Limitations

In addition to the limitations of the primary studies, including the relatively small number of investigations and the high heterogeneity observed across studies, several methodological limitations of this review should be considered when interpreting the findings. First, the tools used to assess burnout, as well as the cut-off scores chosen to identify burnout prevalence, differed significantly across studies. For example, the criteria for “high” burnout varied even between studies using the same version of the Maslach Burnout Inventory. Second, while comparisons were made between economic tiers, less studies were conducted in LMICs, and none in Africa. The lack of investigations conducted in LMICs, particularly across the African continent, could potentially introduce a bias in the comparison between HICs and LMICs. Therefore, the observed subgroup differences may not be representative of all LMIC settings and should not be interpreted as evidence of a systematic income-related difference in burnout prevalence.

Furthermore, many studies involved samples of physiotherapists working in multiple settings (such as combined clinic and hospital staff), meaning that sub-analyses based on different clinical contexts could not be carried out Additionally, the fact that almost all studies failed to define the comparability between those who responded and those who did not might have introduced further selection biases.

Finally, the review is limited by the inclusion of studies from undifferentiated periods of the pandemic, preventing an analysis of differences between specific COVID-19 waves. The exclusion of studies that reported burnout only as a mean level, rather than a prevalence figure, may have also introduced a selection bias into the final pooled estimates.

Publication bias could not be reliably assessed, because tests for funnel-plot asymmetry have limited validity for prevalence data and in the presence of substantial heterogeneity.

## Conclusion

During the COVID-19 pandemic, burnout was frequently reported among physiotherapists, although prevalence estimates varied substantially across studies. The pooled prevalence of overall burnout tends to be lower in HIC studies than in LMIC studies, whereas this pattern was significant only for EE. The interpretation of these findings is limited by substantial between-study heterogeneity and by differences in instruments, cut-off values, and operational definitions of burnout. Further studies using standardized definitions and comparable assessment methods are needed to clarify differences in burnout prevalence across healthcare systems and country income groups.

## Data Availability

All data analyzed in the study are included in the article.
