# Supplementary material for: Prevalence of burnout in physiotherapists during the COVID-19 pandemic: systematic review and meta-analysis
Source: Front Public Health. 2026 Oct 29;14:1978417. doi: 10.3389/fpubh.2026.1978417 (PMC13754879; doi:10.3389/fpubh.2026.1978417)
Supplement: Supplementary file 1 [file Data_Sheet_1.PDF]

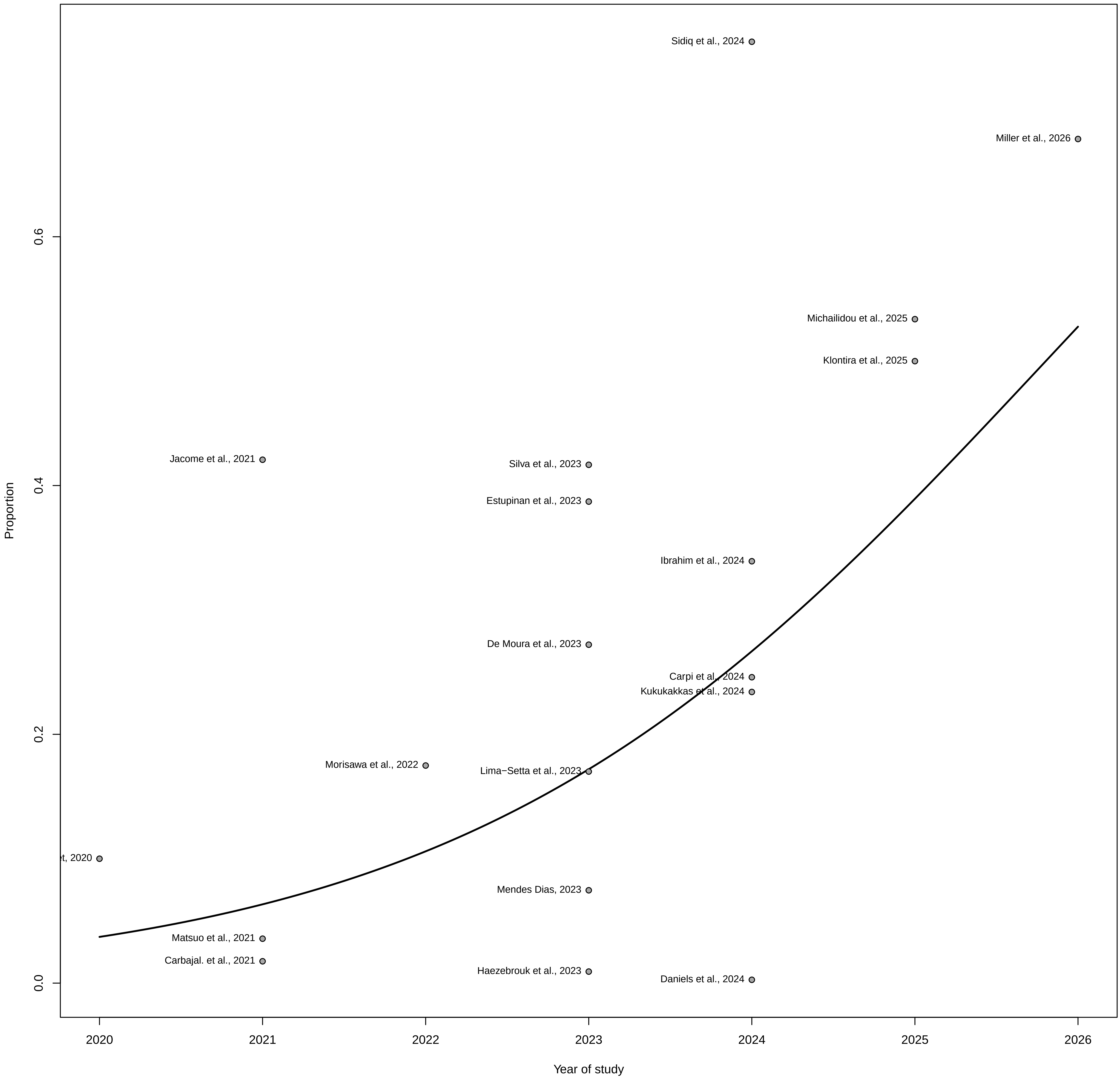

Sidiq et al., 2024
Miller et al., 2026
Michailidou et al., 2025
Klontira et al., 2025
Jacome et al., 2021
Silva et al., 2023
Estupinan et al., 2023
Ibrahim et al., 2024
De Moura et al., 2023
Carpi et al., 2024
Kukukakkas et al., 2024
Morisawa et al., 2022
Lima−Setta et al., 2023
t, 2020
Mendes Dias, 2023
Matsuo et al., 2021
Carbajal. et al., 2021
Haezebrouk et al., 2023
Daniels et al., 2024
0.0
0.2
0.4
0.6
Proportion
2020
2021
2022
2023
2024
2025
2026
Year of study

```
Mixed-Effects Model (k = 19; tau^2 estimator: ML)

  logLik  deviance       AIC       BIC      AICc
-39.8911    6.3450   85.7823   88.6156   87.3823

tau^2 (estimated amount of residual heterogeneity):     2.3915
tau (square root of estimated tau^2 value):             1.5465
I^2 (residual heterogeneity / unaccounted variability): 98.28%
H^2 (unaccounted variability / sampling variability):   58.03

Tests for Residual Heterogeneity:
Wld(df = 17) = 312.3654, p-val < .0001
LRT(df = 17) = 572.0066, p-val < .0001

Test of Moderators (coefficient 2):
QM(df = 1) = 4.6297, p-val = 0.0314

Model Results:

             estimate        se     zval    pval       ci.lb      ci.ub
intrcpt    -1136.4171  527.4998  -2.1543  0.0312  -2170.2978  -102.5365  *
.subgroup      0.5610    0.2607   2.1517  0.0314      0.0500     1.0720  *

---
Signif. codes:  0 '***' 0.001 '**' 0.01 '*' 0.05 '.' 0.1 ' ' 1
```

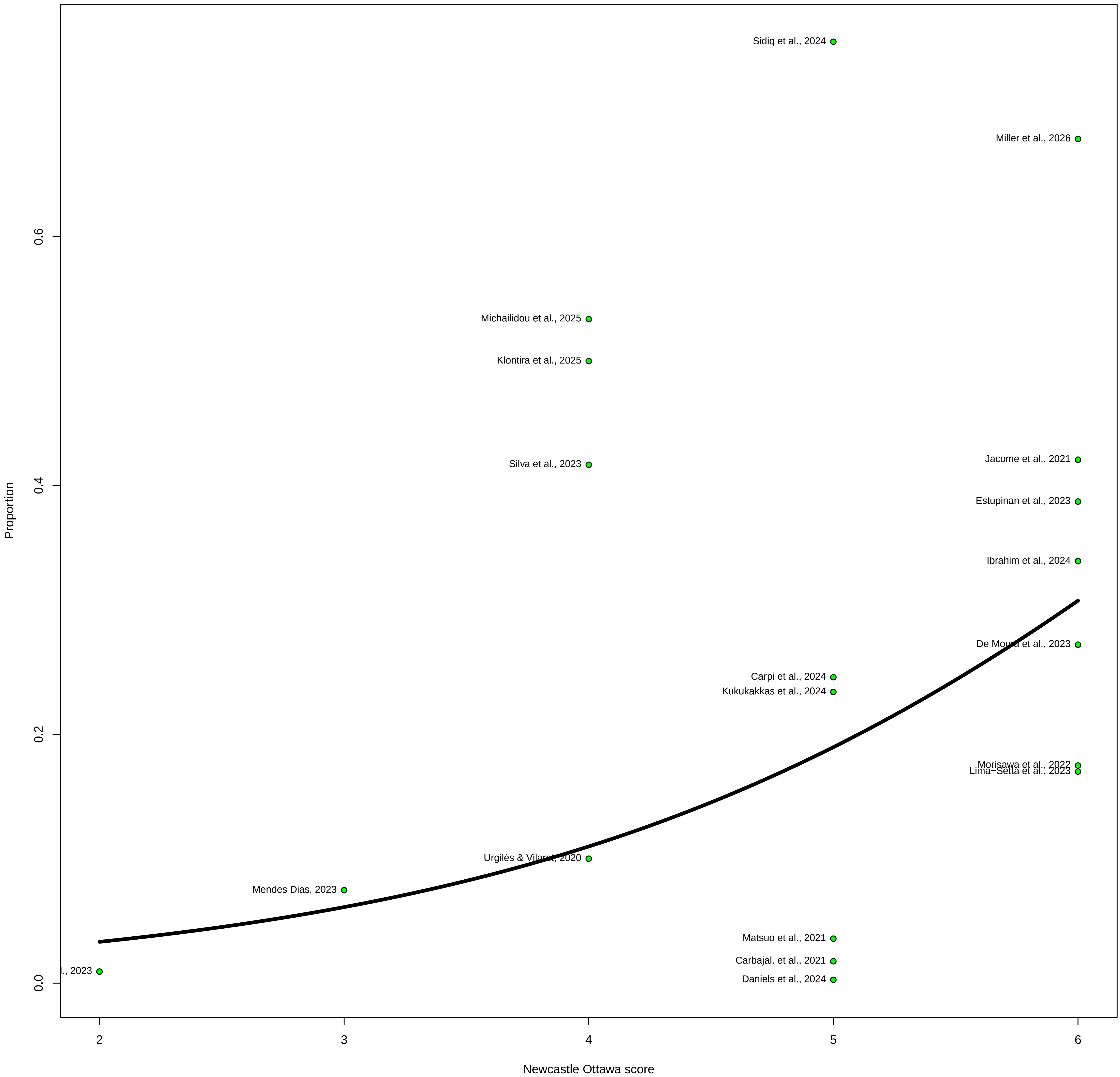

Sidiq et al., 2024
Miller et al., 2026
Michailidou et al., 2025
Klontira et al., 2025
Silva et al., 2023
Jacome et al., 2021
Estupinan et al., 2023
Ibrahim et al., 2024
De Moura et al., 2023
Carpi et al., 2024
Kukukakkas et al., 2024
Morisawa et al., 2022
Lima−Setta et al., 2023
Urgilés & Vilaret, 2020
Mendes Dias, 2023
Matsuo et al., 2021
Carbajal. et al., 2021
Daniels et al., 2024
l., 2023
0.0
0.2
0.4
0.6
Proportion
2
3
4
5
6
Newcastle Ottawa score

```
Mixed−Effects Model (k = 19; tau^2 estimator: ML)

  logLik  deviance       AIC       BIC      AICc
−39.3699    5.3025   84.7398   87.5731   86.3398

tau^2 (estimated amount of residual heterogeneity):     2.7838
tau (square root of estimated tau^2 value):             1.6685
I^2 (residual heterogeneity / unaccounted variability): 98.66%
H^2 (unaccounted variability / sampling variability):   74.56

Tests for Residual Heterogeneity:
Wld(df = 17) = 407.0746, p−val < .0001
LRT(df = 17) = 664.6391, p−val < .0001

Test of Moderators (coefficient 2):
QM(df = 1) = 2.7592, p−val = 0.0967

Model Results:

           estimate      se     zval    pval    ci.lb    ci.ub
intrcpt     −4.6525  1.9772  −2.3531  0.0186  −8.5277  −0.7773  *
.subgroup    0.6401  0.3853   1.6611  0.0967  −0.1152   1.3953  .

−−−
Signif. codes:  0 '***' 0.001 '**' 0.01 '*' 0.05 '.' 0.1 ' ' 1
```

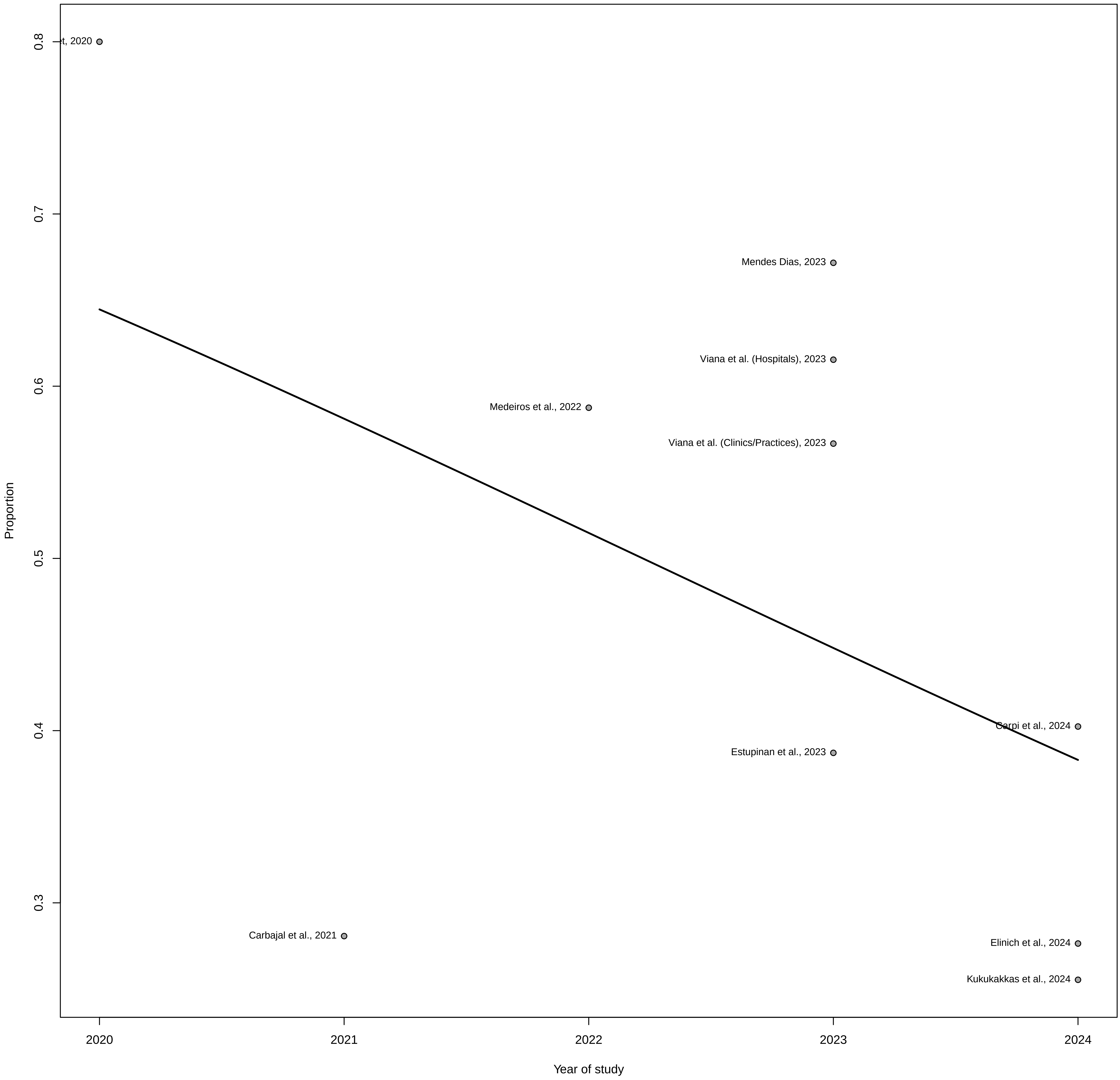

t, 2020
Mendes Dias, 2023
Viana et al. (Hospitals), 2023
Medeiros et al., 2022
Viana et al. (Clinics/Practices), 2023
Carpi et al., 2024
Estupinan et al., 2023
Carbajal et al., 2021
Elinich et al., 2024
Kukukakkas et al., 2024
0.8
0.7
0.6
0.5
0.4
0.3
Proportion
2020
2021
2022
2023
2024
Year of study

```
Mixed-Effects Model (k = 10; tau^2 estimator: ML)

  logLik  deviance       AIC       BIC      AICc
-22.7565    2.2811   51.5130   52.4207   55.5130

tau^2 (estimated amount of residual heterogeneity):     0.3375
tau (square root of estimated tau^2 value):             0.5810
I^2 (residual heterogeneity / unaccounted variability): 85.28%
H^2 (unaccounted variability / sampling variability):   6.79

Tests for Residual Heterogeneity:
Wld(df = 8) = 50.5312, p-val < .0001
LRT(df = 8) = 54.8565, p-val < .0001

Test of Moderators (coefficient 2):
QM(df = 1) = 2.1536, p-val = 0.1422

Model Results:

           estimate        se     zval    pval      ci.lb      ci.ub
intrcpt    542.0580  369.4901   1.4670  0.1424  -182.1293  1266.2453
.subgroup   -0.2681    0.1827  -1.4675  0.1422    -0.6260     0.0899

---
Signif. codes:  0 '***' 0.001 '**' 0.01 '*' 0.05 '.' 0.1 ' ' 1
```

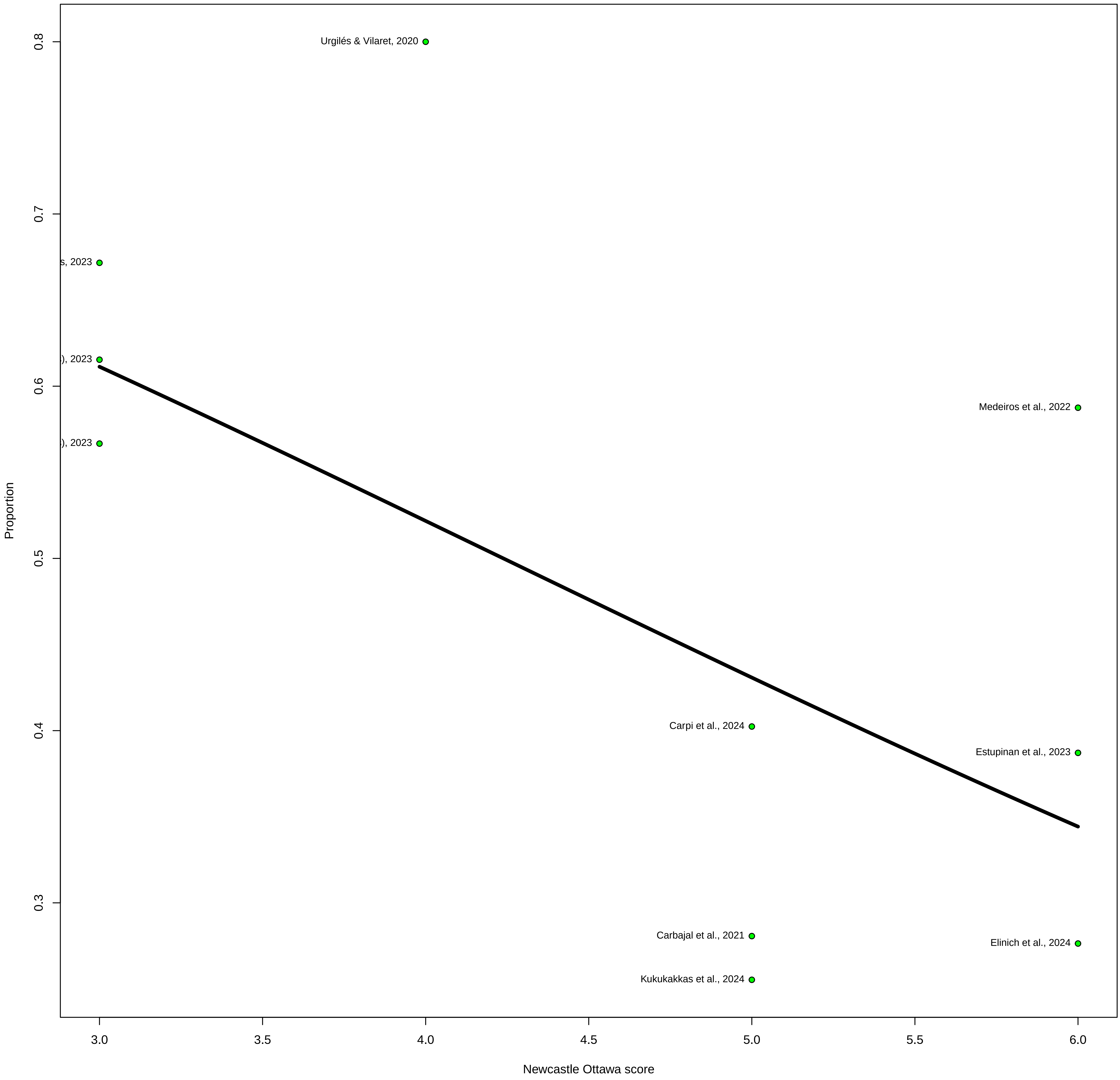

Urgilés & Vilaret, 2020
s, 2023
), 2023
), 2023
Medeiros et al., 2022
Carpi et al., 2024
Estupinan et al., 2023
Carbajal et al., 2021
Elinich et al., 2024
Kukukakkas et al., 2024
0.3
0.4
0.5
0.6
0.7
0.8
Proportion
3.0
3.5
4.0
4.5
5.0
5.5
6.0
Newcastle Ottawa score

```
Mixed-Effects Model (k = 10; tau^2 estimator: ML)

  logLik  deviance       AIC       BIC      AICc
-23.2756    3.3193   52.5512   53.4590   56.5512

tau^2 (estimated amount of residual heterogeneity):     0.2245
tau (square root of estimated tau^2 value):             0.4738
I^2 (residual heterogeneity / unaccounted variability): 81.12%
H^2 (unaccounted variability / sampling variability):   5.30

Tests for Residual Heterogeneity:
Wld(df = 8) = 38.8157, p-val < .0001
LRT(df = 8) = 40.8677, p-val < .0001

Test of Moderators (coefficient 2):
QM(df = 1) = 5.6132, p-val = 0.0178

Model Results:

           estimate      se     zval    pval    ci.lb    ci.ub
intrcpt      1.5498  0.7588   2.0425  0.0411   0.0626   3.0371  *
.subgroup   -0.3657  0.1543  -2.3692  0.0178  -0.6682  -0.0632  *

---
Signif. codes:  0 '***' 0.001 '**' 0.01 '*' 0.05 '.' 0.1 ' ' 1
```

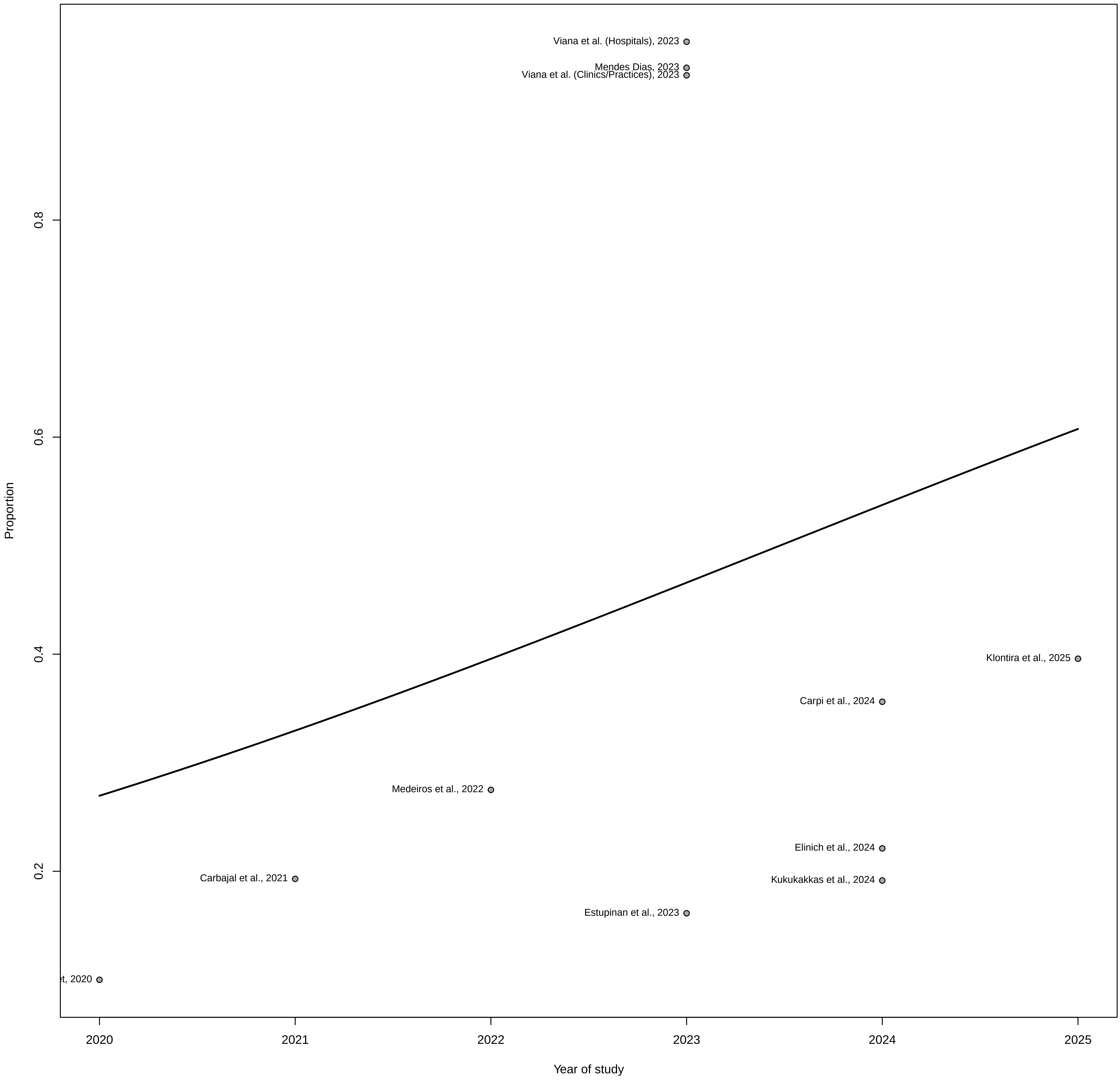
Viana et al. (Hospitals), 2023
Mendes Dias, 2023
Viana et al. (Clinics/Practices), 2023
Klontira et al., 2025
Carpi et al., 2024
Medeiros et al., 2022
Elinich et al., 2024
Carbajal et al., 2021
Kukukakkas et al., 2024
Estupinan et al., 2023
t, 2020
0.8
0.6
0.4
0.2
Proportion
2020
2021
2022
2023
2024
2025
Year of study

```
Mixed−Effects Model (k = 11; tau^2 estimator: ML)

  logLik  deviance       AIC       BIC      AICc
−21.1272    2.2675   48.2545   49.4481   51.6830

tau^2 (estimated amount of residual heterogeneity):     3.3701
tau (square root of estimated tau^2 value):             1.8358
I^2 (residual heterogeneity / unaccounted variability): 97.81%
H^2 (unaccounted variability / sampling variability):   45.57

Tests for Residual Heterogeneity:
Wld(df = 9) = 92.1456, p−val < .0001
LRT(df = 9) = 217.3278, p−val < .0001

Test of Moderators (coefficient 2):
QM(df = 1) = 0.4505, p−val = 0.5021

Model Results:

           estimate        se     zval    pval       ci.lb      ci.ub
intrcpt    −580.2225  864.2389  −0.6714  0.5020  −2274.0996  1113.6546
.subgroup     0.2867    0.4272   0.6712  0.5021     −0.5506     1.1241

−−−
Signif. codes:  0 '***' 0.001 '**' 0.01 '*' 0.05 '.' 0.1 ' ' 1
```

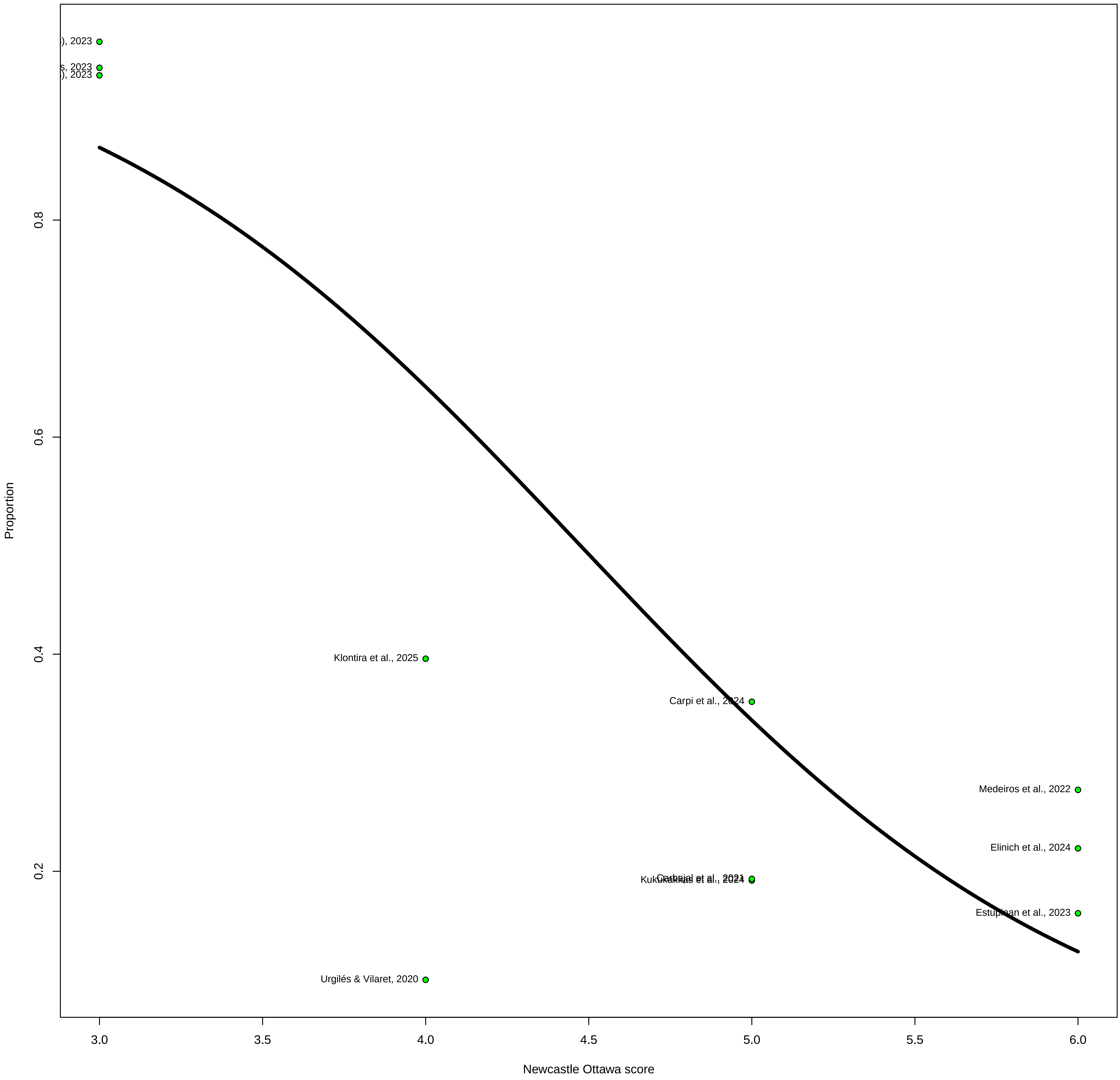

), 2023
s, 2023
), 2023
Klontira et al., 2025
Carpi et al., 2024
Medeiros et al., 2022
Elinich et al., 2024
Estupiñan et al., 2023
Urgilés & Vilaret, 2020
0.2
0.4
0.6
0.8
Proportion
3.0
3.5
4.0
4.5
5.0
5.5
6.0
Newcastle Ottawa score

```
Mixed−Effects Model (k = 11; tau^2 estimator: ML)

  logLik  deviance       AIC       BIC      AICc
−22.3605    4.7340   50.7210   51.9147   54.1496

tau^2 (estimated amount of residual heterogeneity):     0.7961
tau (square root of estimated tau^2 value):             0.8922
I^2 (residual heterogeneity / unaccounted variability): 91.30%
H^2 (unaccounted variability / sampling variability):   11.50

Tests for Residual Heterogeneity:
Wld(df = 9) = 41.4066, p−val < .0001
LRT(df = 9) = 61.7796, p−val < .0001

Test of Moderators (coefficient 2):
QM(df = 1) = 20.7476, p−val < .0001

Model Results:

           estimate      se     zval    pval    ci.lb    ci.ub
intrcpt      5.6835  1.3503   4.2090  <.0001   3.0369   8.3301  ***
.subgroup   −1.2701  0.2788  −4.5550  <.0001  −1.8166  −0.7236  ***

−−−
Signif. codes:  0 '***' 0.001 '**' 0.01 '*' 0.05 '.' 0.1 ' ' 1
```

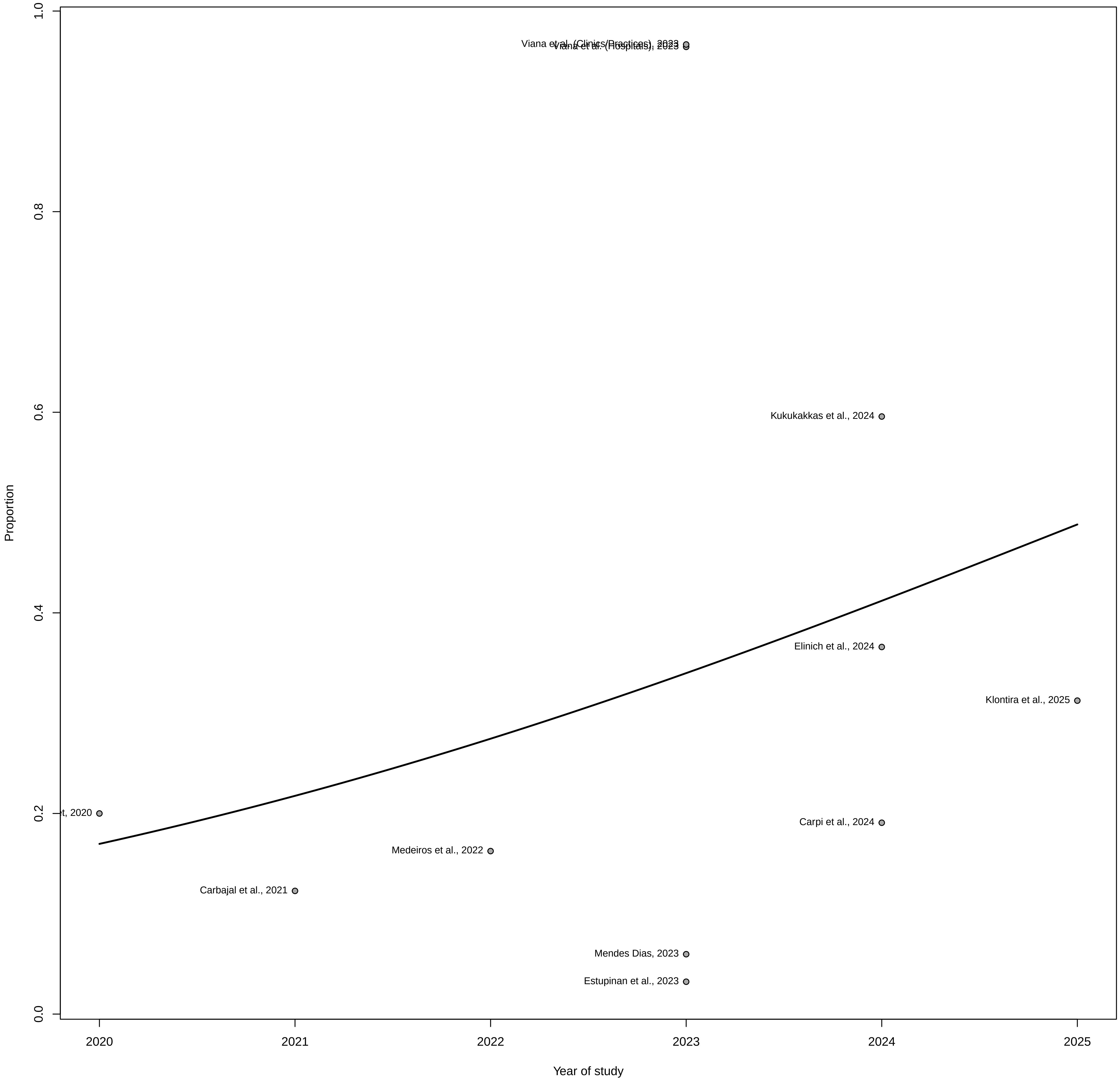

Viana et al. (Clinics/Practices), 2023
Viana et al. (Hospitals), 2023
Kukukakkas et al., 2024
Elinich et al., 2024
Klontira et al., 2025
t, 2020
Carpi et al., 2024
Medeiros et al., 2022
Carbajal et al., 2021
Mendes Dias, 2023
Estupinan et al., 2023
Proportion
0.0
0.2
0.4
0.6
0.8
1.0
2020
2021
2022
2023
2024
2025
Year of study

```
Mixed−Effects Model (k = 11; tau^2 estimator: ML)

  logLik  deviance       AIC       BIC      AICc
−20.3072    2.5791   46.6143   47.8080   50.0429

tau^2 (estimated amount of residual heterogeneity):     3.9413
tau (square root of estimated tau^2 value):             1.9853
I^2 (residual heterogeneity / unaccounted variability): 98.07%
H^2 (unaccounted variability / sampling variability):   51.92

Tests for Residual Heterogeneity:
Wld(df = 9) = 84.8840, p−val < .0001
LRT(df = 9) = 193.5781, p−val < .0001

Test of Moderators (coefficient 2):
QM(df = 1) = 0.4633, p−val = 0.4961

Model Results:

           estimate        se     zval    pval       ci.lb      ci.ub
intrcpt    −623.8947  915.6390  −0.6814  0.4956  −2418.5142  1170.7248
.subgroup     0.3081    0.4526   0.6806  0.4961     −0.5791     1.1952

−−−
Signif. codes:  0 '***' 0.001 '**' 0.01 '*' 0.05 '.' 0.1 ' ' 1
```

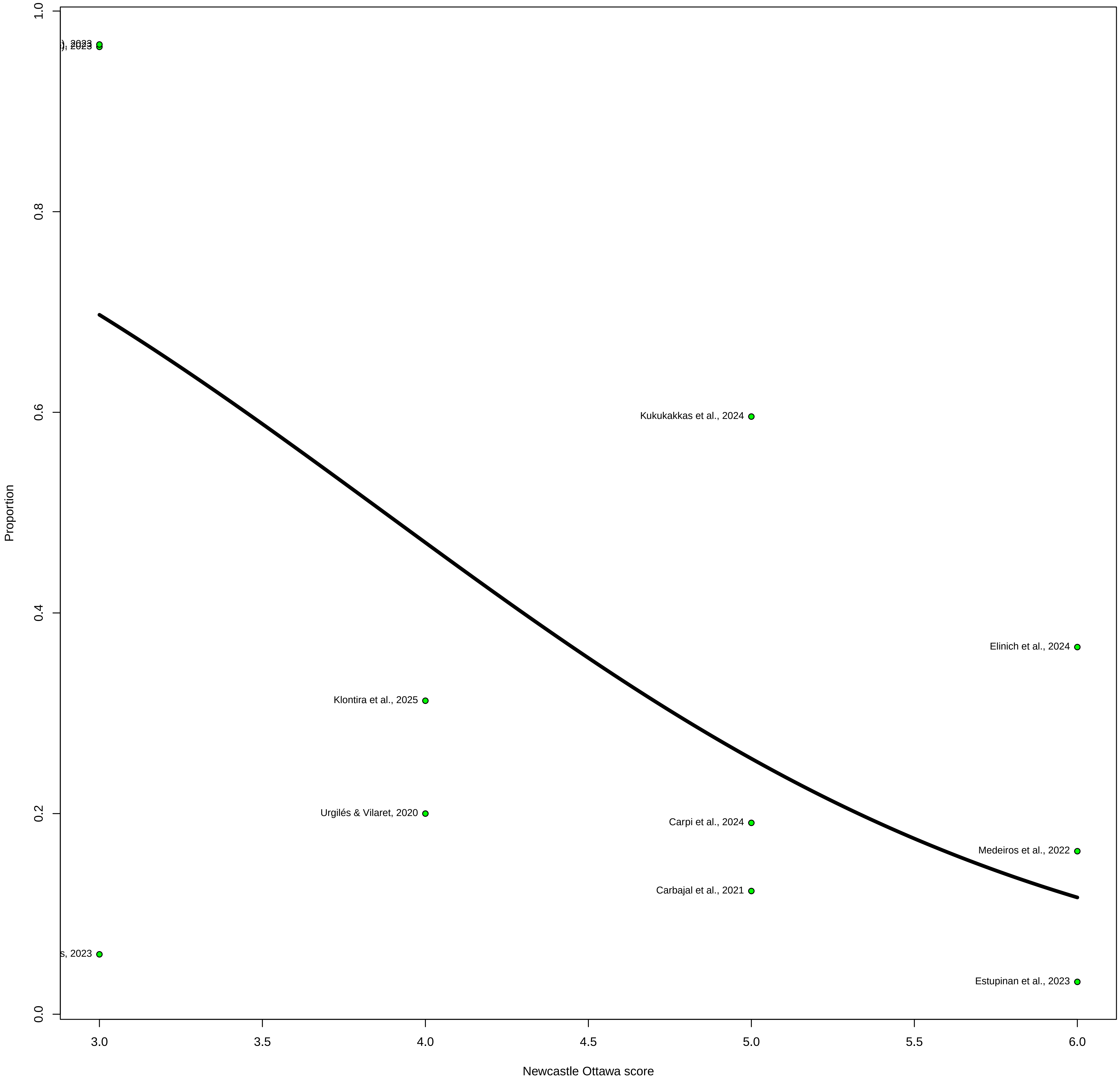
Proportion
Newcastle Ottawa score
1.0
0.8
0.6
0.4
0.2
0.0
3.0
3.5
4.0
4.5
5.0
5.5
6.0
Kukukakkas et al., 2024
Elinich et al., 2024
Klontira et al., 2025
Urgilés & Vilaret, 2020
Carpi et al., 2024
Medeiros et al., 2022
Carbajal et al., 2021
s, 2023
Estupinan et al., 2023

```
Mixed-Effects Model (k = 11; tau^2 estimator: ML)

  logLik  deviance       AIC       BIC      AICc
-20.0799    2.1246   46.1599   47.3535   49.5884

tau^2 (estimated amount of residual heterogeneity):     3.1559
tau (square root of estimated tau^2 value):             1.7765
I^2 (residual heterogeneity / unaccounted variability): 97.32%
H^2 (unaccounted variability / sampling variability):   37.25

Tests for Residual Heterogeneity:
Wld(df = 9) = 90.0189, p-val < .0001
LRT(df = 9) = 192.0032, p-val < .0001

Test of Moderators (coefficient 2):
QM(df = 1) = 3.4948, p-val = 0.0616

Model Results:

           estimate      se     zval    pval    ci.lb   ci.ub
intrcpt      3.6949  2.4265   1.5227  0.1278  -1.0610  8.4507
.subgroup   -0.9536  0.5101  -1.8694  0.0616  -1.9534  0.0462  .

---
Signif. codes:  0 '***' 0.001 '**' 0.01 '*' 0.05 '.' 0.1 ' ' 1
```
