# Supplementary material for: Prevalence of burnout in physiotherapists during the COVID-19 pandemic: systematic review and meta-analysis
Source: Front Public Health. 2026 Oct 29;14:1978417. doi: 10.3389/fpubh.2026.1978417 (PMC13754879; doi:10.3389/fpubh.2026.1978417)
Supplement: Supplementary file 2 [file Data_Sheet_2.PDF]

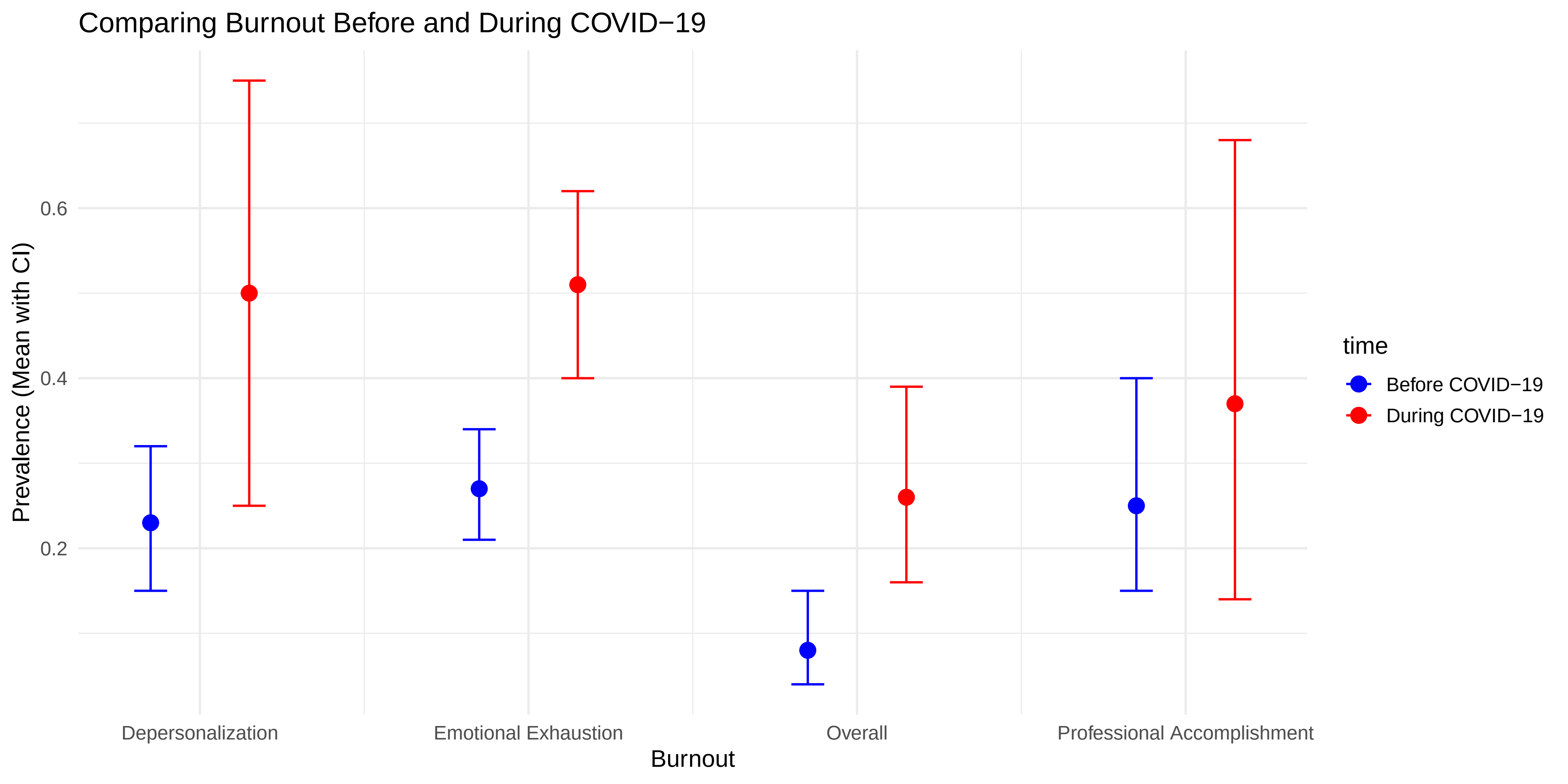
Comparing Burnout Before and During COVID−19
Prevalence (Mean with CI)
0.6
0.4
0.2
Depersonalization
Emotional Exhaustion
Overall
Professional Accomplishment
Burnout
time
Before COVID−19
During COVID−19

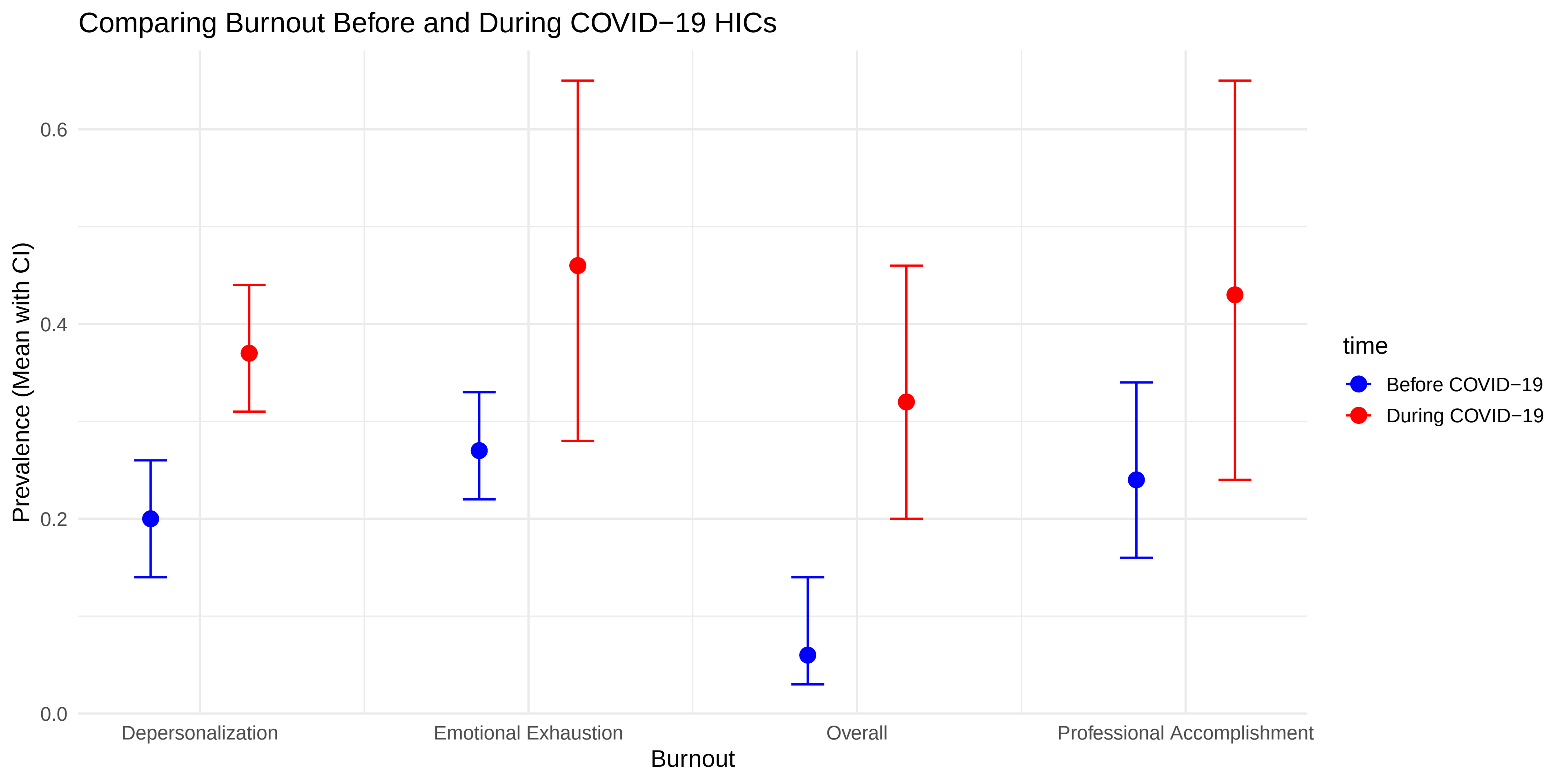
Comparing Burnout Before and During COVID−19 HICs
Prevalence (Mean with CI)
0.6
0.4
0.2
0.0
Depersonalization
Emotional Exhaustion
Overall
Professional Accomplishment
Burnout
time
Before COVID−19
During COVID−19

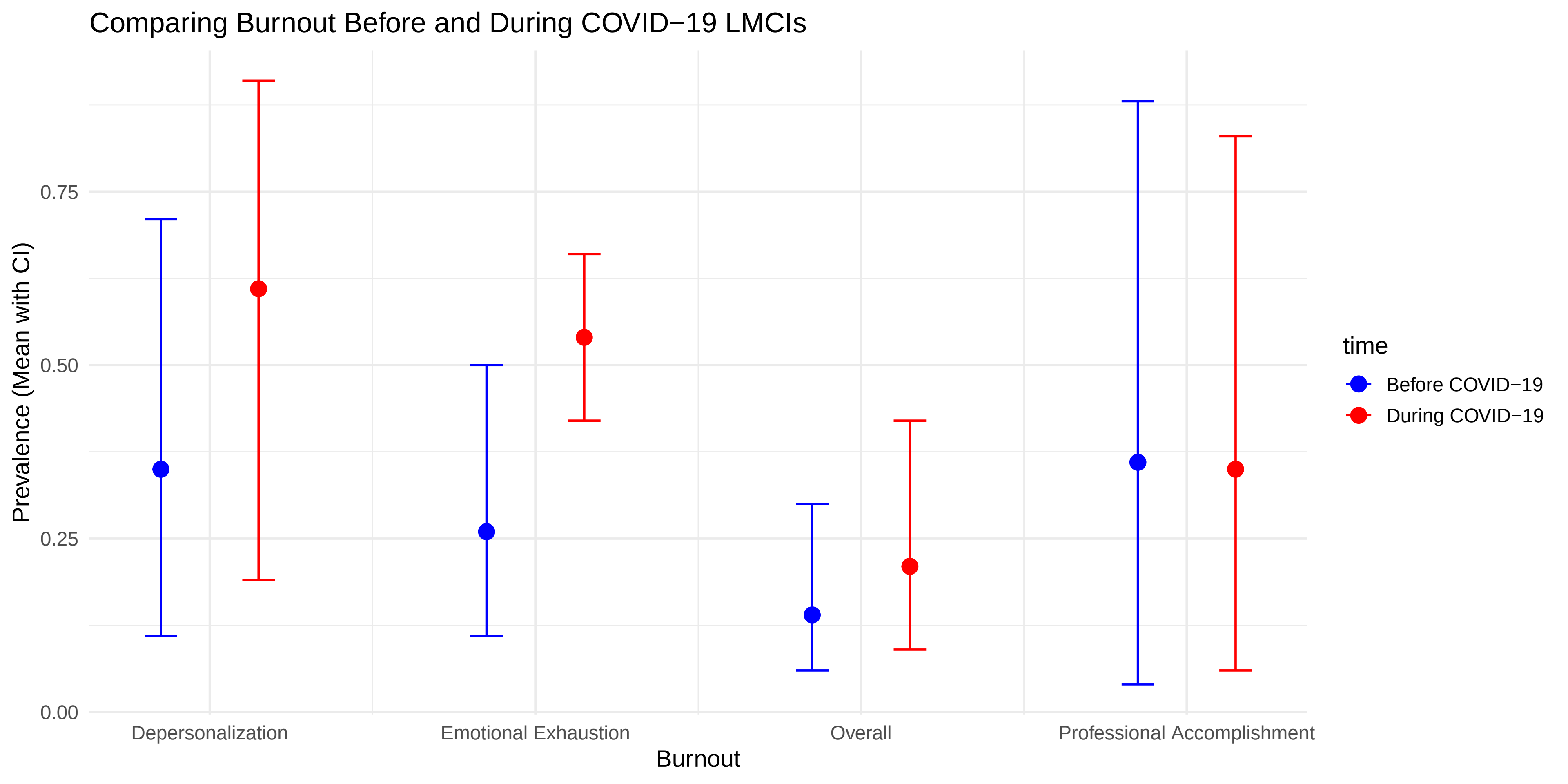
Comparing Burnout Before and During COVID−19 LMCIs
Prevalence (Mean with CI)
0.75
0.50
0.25
0.00
Depersonalization
Emotional Exhaustion
Overall
Professional Accomplishment
Burnout
time
Before COVID−19
During COVID−19
