# Supplementary material for: Prevalence of burnout in physiotherapists during the COVID-19 pandemic: systematic review and meta-analysis
Source: Front Public Health. 2026 Oct 29;14:1978417. doi: 10.3389/fpubh.2026.1978417 (PMC13754879; doi:10.3389/fpubh.2026.1978417)
Supplement: Supplementary file 3 [file Supplementary_file_1.DOCX]

**Search strategy (PubMed)**

(burnout OR (burned AND out) OR depersonalization OR (emotional AND exhaus-tion) OR 'professional burnout' OR 'professional burnout' OR 'emotional stress' OR 'emotional stress' OR 'psychological stress' OR 'psychological stress' OR (psycholog-ical AND stress) OR ('compassion fatigue' OR 'compassion fatigue'))

AND

((cohort AND design) OR (cohort AND stud*) OR ('cross sectional' AND analysis) OR ('cross sectional' AND stud*) OR (epidemiologic AND stud*) OR incidence OR (longitudi-nal AND design) OR (longitudinal AND stud*) OR (observational AND stud*) OR (population AND stud*) OR prevalence OR (prospective AND design) OR (prospec-tive AND stud*) OR (retrospective AND design) OR (retrospective AND stud*))

AND

(‘physical ther*’ OR physiother* OR ‘Rehabilitation ther*’)

AND

(SARS-CoV-2 OR COVID-19 OR SARS; 2019-nCoV OR coronavirus)
